# Calcium phosphate precipitation inhibits mitochondrial energy metabolism

**DOI:** 10.1371/journal.pcbi.1006719

**Published:** 2019-01-07

**Authors:** Sathyavani Malyala, Yizhu Zhang, Jasiel O. Strubbe, Jason N. Bazil

**Affiliations:** Department of Physiology, Michigan State University, East Lansing, MI, United States of America; Virginia Polytechnic Institute and State University, UNITED STATES

## Abstract

Early studies have shown that moderate levels of calcium overload can cause lower oxidative phosphorylation rates. However, the mechanistic interpretations of these findings were inadequate. And while the effect of excessive calcium overload on mitochondrial function is well appreciated, there has been little to no reports on the consequences of low to moderate calcium overload. To resolve this inadequacy, mitochondrial function from guinea pig hearts was quantified using several well-established methods including high-resolution respirometry and spectrofluorimetry and analyzed using mathematical modeling. We measured key mitochondrial variables such as respiration, mitochondrial membrane potential, buffer calcium, and substrate effects for a range of mitochondrial calcium loads from near zero to levels approaching mitochondrial permeability transition. In addition, we developed a computer model closely mimicking the experimental conditions and used this model to design experiments capable of eliminating many hypotheses generated from the data analysis. We subsequently performed those experiments and determined why mitochondrial ADP-stimulated respiration is significantly lowered during calcium overload. We found that when calcium phosphate levels, not matrix free calcium, reached sufficient levels, complex I activity is inhibited, and the rate of ATP synthesis is reduced. Our findings suggest that calcium phosphate granules form physical barriers that isolate complex I from NADH, disrupt complex I activity, or destabilize cristae and inhibit NADH-dependent respiration.

## Author summary

Mitochondrial calcium handling has been studied for nearly half a century. As we understand it today, low concentrations (1–10 nmol/mg mitochondria) of calcium exert beneficial effects on energy transduction. And high concentrations (>500 nmol/mg mitochondria) lead to respiratory uncoupling and membrane damage. But relatively little is known about the effect of moderate calcium concentrations (10–500 nmol/mg mitochondria) on mitochondrial function. At these concentrations, mitochondrial membrane integrity remains intact and energized, while ATP synthesis becomes significantly impaired. Prior studies have postulated several possible mechanisms, but the precise consequence of calcium overload on mitochondrial ATP production remained obscure. In this study, we combine experimental and computational approaches to show that calcium phosphate precipitation, as opposed to matrix free calcium, inhibits respiratory function at complex I just enough to limit proton pumping during oxidative phosphorylation and decrease ATP synthesis rates.

## Introduction

The heart is one of the most energy dependent tissues in the body and requires a very high rate of ATP synthesis and oxygen delivery. Energy stored in carbon fuels is extracted by a network of biochemical reactions to produce reducing equivalents such as NADH and UQH_2_. These reducing equivalents feed electrons into the electron transport system to generate a proton electrochemical gradient which is then utilized to synthesize ATP. This entire process is tightly regulated and maintains stable levels of ATP despite rapid changes in workload and energy demand [1]. But when the heart is subjected to stressors, such as ischemia, this tight coupling quickly becomes destabilized.

Rhythmic cytosolic calcium signals characterized by spike-like transients occur during normal physiological function in the heart. These calcium transients increase peak cytosolic calcium levels from approximately 100 nM up to levels ranging from 500 nM to 1 μM, depending on energy demand [2]. These transients can result in net calcium accumulation by mitochondria via the calcium uniporter complex [3, 4] and cause matrix calcium levels to increase from 100 nM to 1 μM and stimulate NADH and ATP production rates [5–7]. To prevent the accumulation of toxic levels of calcium, the mitochondrial sodium calcium exchanger (NCLX) [3, 8, 9] removes one matrix calcium ion in exchange for three cytosolic sodium ions in an electrogenic exchange mechanism. Under ischemic conditions, cytosolic calcium levels can rise up to 3 μM [10]. At these high levels of cytosolic calcium, calcium uptake exceeds calcium efflux [11]. This can translate into massive increases in mitochondrial calcium content reaching up to 1 M total calcium stored as calcium phosphate granules [12–15]. In this calcium overloaded state, the ATP production capacity of mitochondria is severely compromised [16], and mitochondria are primed for a devastating injury upon reperfusion known as mitochondrial permeability transition [17–19].

The exact mechanism behind the reduction in mitochondrial ATP production rates caused by calcium overload is unknown. Some have argued that calcium overload results in a direct inhibition of complex I [20, 21]. This is supported by data that show complex I activity is reduced after reperfusion injury [22]. Others have reported that calcium overload inhibits the matrix dehydrogenase complexes pyruvate dehydrogenase and alpha-ketoglutarate dehydrogenase [23–25]. In addition to these reports, others have claimed calcium overload directly inhibits the adenine nucleotide translocase [26], reduces the availability of free and/or Mg-bound ADP for oxidative phosphorylation and transport caused by calcium chelation [27, 28], causes net loss of matrix purine nucleotides [29], or lowers the membrane potential for ATP production [30]. Another study points towards inhibition of cytochrome c oxidase by high levels of calcium [31]. Calcium phosphate granules have also been implicated in the inhibition of oxidative phosphorylation [32]. Yet, in other studies, mitochondrial calpains have been suspected to play a major role in calcium-induced mitochondrial dysfunction [33, 34]. Needless to say, the cause of impaired mitochondrial function due to calcium overload still remains uncertain.

In this study, we utilize a joint experimental and computational approach to identify the likely mechanisms and gain physiological insight into the effects of calcium overload on mitochondrial function and ATP production capacity. We tested the effect of low to moderate calcium overload conditions on ADP-stimulated respiration and membrane energization. The levels tested were below the threshold sufficient to cause mitochondrial permeability transition and demonstrate a permeabilization-independent effect of excess calcium on mitochondrial function. We found that calcium chelation or depressed membrane potential during sodium/calcium cycling did not lead to lower rates of oxidative phosphorylation. In addition, we did not detect any loss of purine nucleotides or direct inhibition of either the adenine nucleotide translocase or cytochrome c oxidase. In addition, our results show that mitochondrial calpains are not the cause of this phenomenon. Our data support the idea that complex I inhibition is the most probable cause of the observed decrease in rates of oxidative phosphorylation. And it is the amount of calcium phosphates accumulated by mitochondria, not the matrix free calcium concentration, that is the key determinant of this inhibition.

## Results

**Fig 1:**
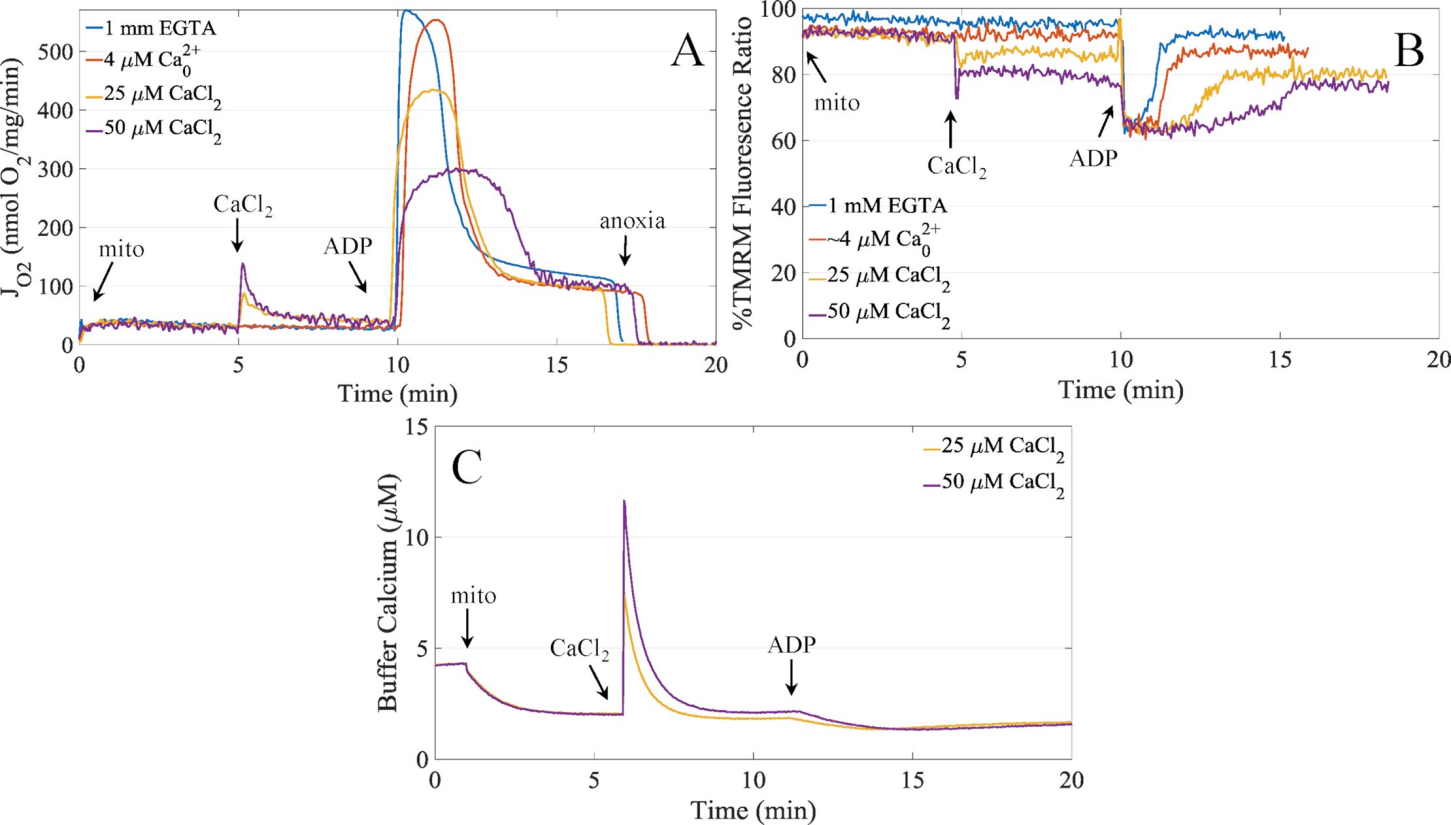
Representative respiration, membrane potential and calcium uptake dynamics. A) Mitochondrial respiration rates demonstrating the calcium-dependent inhibition of ADP-stimulated respiration. B) Corresponding membrane potential values inferred from TMRM fluorescence quenching. Data are cleaned of injection artifacts and calibration portions. C) Corresponding calcium uptake dynamics for the 25 μM and 50 μM CaCl_2_ boluses. Experimental conditions are given in the Methods.

The effects of calcium on mitochondrial energetics span from the beneficial in the low range (10’s of nmol/mg) to the catastrophic in the high range (greater than 500 nmol/mg). In the low range, matrix calcium activates TCA cycle dehydrogenases and other matrix enzymes that increase the rate of proton pumping and ATP synthesis. In the high range, calcium overload leads to mitochondrial permeability transition and membrane rupture. In this state, mitochondria are unable to maintain a proton electrochemical gradient and thus unable to generate ATP. However, in the intermediate range, calcium leads to a depression in ATP synthesis rates, despite maintaing membrane integrity and being able to maintain a proton electrochemical gradient. Fig 1A shows the prototypical response of isolated mitochondrial respiration to varying amounts of calcium exposure. As the amount of calcium taken up by the mitochondria increases, the ADP-stimulated respiration rates are lowered in a titratable manner. In these experiments, ATP synthesis remains coupled to oxygen consumption. The exact cause of this reduction in ATP synthesis rates in calcium loaded mitochondria is not precisely known. Herein, we test a combination of experimentally and computationally derived hypotheses to determine why ATP synthesis is inhibited in the calcium overloaded state.

### Experimental approach

Based on the data shown in Fig 1, the following mechanisms explain the calcium inhibition data: i) lower membrane potential caused by sodium/calcium cycling lowers the driving force for ATP production; ii) a subpopulation of mitochondria permeabilize and reduce the total number of mitochondria capable of synthesizing ATP; iii) calpain activation leads to proteolytic loss of electron transport and/or ATP synthesis function; iv) available ADP and/or phosphate for phosphorylation is depleted or reduced by calcium binding, incorporation into calcium phosphate granules, or net loss; and iv) direct inhibition of calcium on mitochondrial processes critical for ATP production. We employed both experimental and computational strategies to test these hypotheses.

The first hypotheses states that the lower membrane potential caused by sodium/calcium cycling lower the thermodynamic driving force, and hence, the rate for ATP synthesis. And while the membrane potential, as shown in Fig 1B, is lower after the CaCl_2_ bolus was given due to sodium/calcium cycling, the membrane potential during ATP synthesis is the same for all calcium conditions. This rules out that calcium-dependent reduction in respiration rates during ADP-stimulated respiration is due to a lowering driving force for ATP production. Thus, hypothesis i) is disproved.

The second hypothesis involves subpopulations of mitochondria responding differently to the calcium challenges. Isolated mitochondria in suspension do not undergo calcium-dependent permeabilization all at once. It is believed that the individual calcium tolerance of each mitochondrion in a population of suspended mitochondria falls within a distribution [35], so that the more susceptible mitochondria undergo permeability transition first. Upon permeabilization, they release their calcium content which is taken up by more calcium-resistant mitochondria until they reach their calcium limit, and so on. This cascade of events might explain the data given in Fig 1A. However, both the membrane potential data in Fig 1B and the calcium uptake data in Fig 1C dispute this notion. The membrane potential data reveal that energized status of the population of mitochondria is stable, and the calcium uptake data show that calcium uptake is robust with no detectable permeabilization. This disproves hypothesis ii).

**Fig 2:**
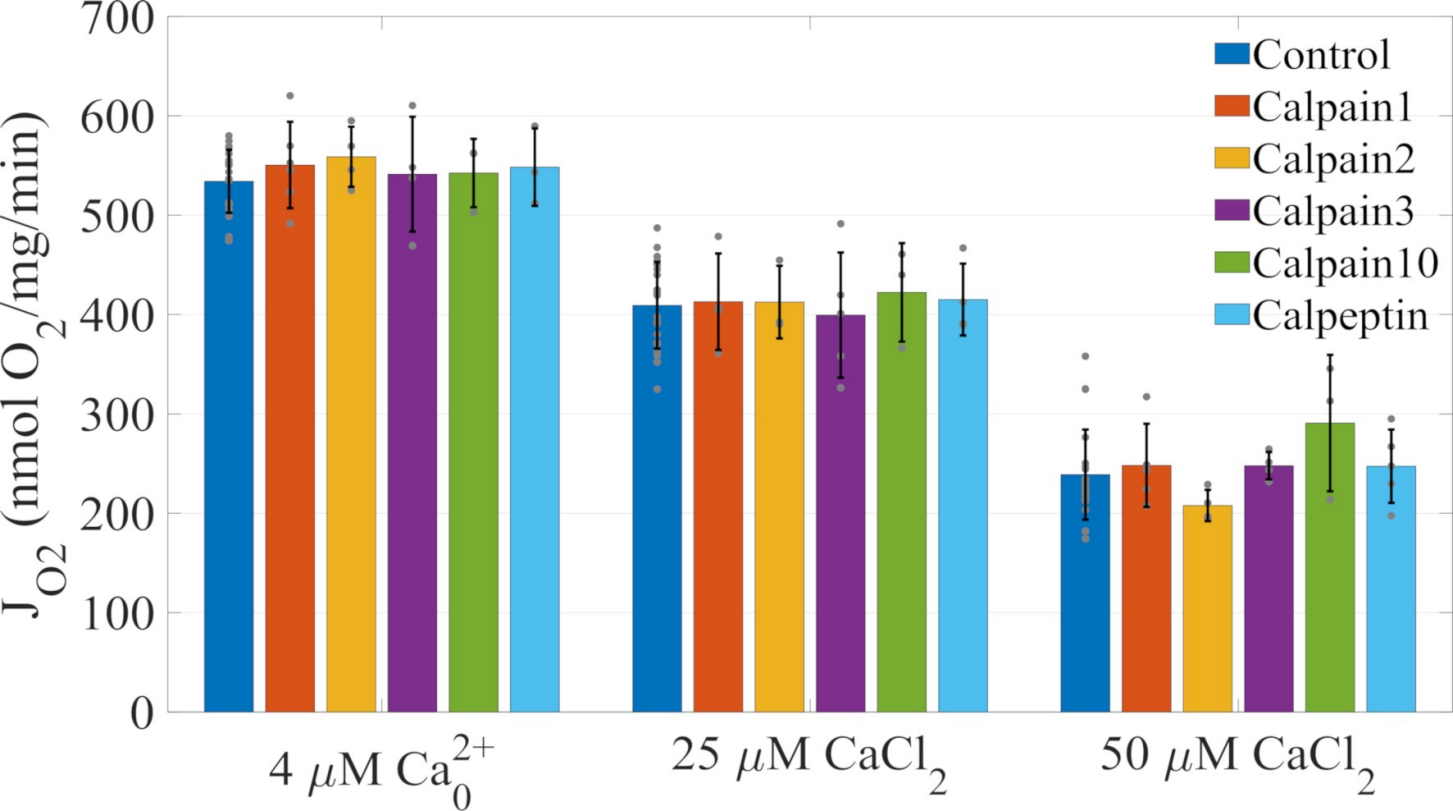
Calcium inhibition on ADP-stimulated respiration is independent of calpain activity. Conditions are like those given in Fig 1. Briefly, 0.1 mg/ml mitochondria are incubated in the presence of 10 μM calpain inhibitor and substrates for five minutes before a bolus of CaCl_2_ is added followed by bolus of 500 μM ADP five minutes after the CaCl_2_ bolus. Calpain inhibitors show no effect on the calcium-dependent inhibition of ADP-stimulated respiration. In all experiments, data are presented as mean +/- standard deviation from three or more biological replicates. Individual data are presented as gray dots. There were no statistically significant differences within calcium treatment groups at a 0.05 alpha level. Control data for each calpain inhibitor were combined.

The next hypothesis argues that calpain activation is the reason why calcium lowers ADP-stimulated respiration. Calpains are intracellular calcium-activated cysteine proteases present in nearly all vertebrate cells [36]. They are often, but not always, associated with subcellular organelles. The physiological function of calpains are not fully understood but they likely play major roles during autolysis. Calpains remain inactive until calcium increases to sufficient levels. In the ischemic myocyte, the dysregulation of cytosolic and mitochondrial calcium is thought to be central to calpain activation. Studies have shown that mitochondrial calpain activation does cleave specific sites on complex I and ATP synthase which has been linked to poor substrate oxidation and ATP production [33, 34, 37]. So we decided to test whether or not calpains played any sort of role in the calcium-induced depression of ADP-stimulated respiration. We experimentally tested this hypothesis by incubating isolated mitochondria with calpain inhibitors prior to calcium exposure. If calpains were responsible for this phenomenon, then inhibiting their proteolytic activity should rescue the observed calcium triggered mitochondrial dysfunction. Our results shown in Fig 2 demonstrate that calpain inhibitors do not relieve this inhibition and thus are not involved in the observed phenomenon. Longer incubation times did not improve mitochondrial function (S1 File). Hypothesis iii) is disproved.

### Computational approach

In order to test the remaining hypotheses, we resorted to utilizing a computational approach. We started with the bioenergetics model from Bazil et al. [38] and added calcium handling, matrix calcium buffering, and updated a few other reactions to improve fits to the original data [39]. See the Supporting Information (S1 File) for details. In brief, very simple mitochondrial calcium uniporter (MCU) and sodium calcium exchanger (NCLX) rate expressions were used with the sodium hydrogen exchanger rate expression from Bazil et al. [40]. We first started with more biophysically detailed MCU [41] and NCLX [42] rate expressions; however, we encountered several issues which precluded us from incorporating these more detailed expressions into the current model. First, the detailed MCU rate expression showed saturation with respect to respiration. This aberrant behavior was due to a calcium dissociation constant for the MCU that was too low causing the calcium current to saturate and produce a shark fin like respiratory dynamic. From Fig 1A, it can clearly be seen no such saturation-like respiratory response is evident. As such, we used a much simpler rate expression with a higher V_max_ and K_D_ for calcium that is more in line with the known characteristics of the channel [43]. In addition to the MCU, the detailed NCLX flux expression was overly complex and prevented adequate fits to the data. In this case, the steep proton dependency was determined to be the main problem. As with the MCU problem, a simpler rate expression was adequate to fit the experimental data. We also included the model of the mitochondrial calcium sequestration system based on data from Blomeyer et al. [44] described in detail below. The last major changes to the model was to the lumped TCA cycle rate expression and updating the complex III rate expression. We noticed the rate expression published with the original model failed to adequately simulate the respiratory behavior after calcium loading. This was due to calcium-dependent alkalization of the matrix not being properly compensated for in the expression and was alleviated by slightly altering the NADH feedback component. In addition to changing the TCA cycle rate expression, we replaced the complex III rate expression with a new one that includes dimer functionality from Bazil et al. [45]. The new rate expressions are given in the Supporting Information (S1 File).

**Fig 3:**
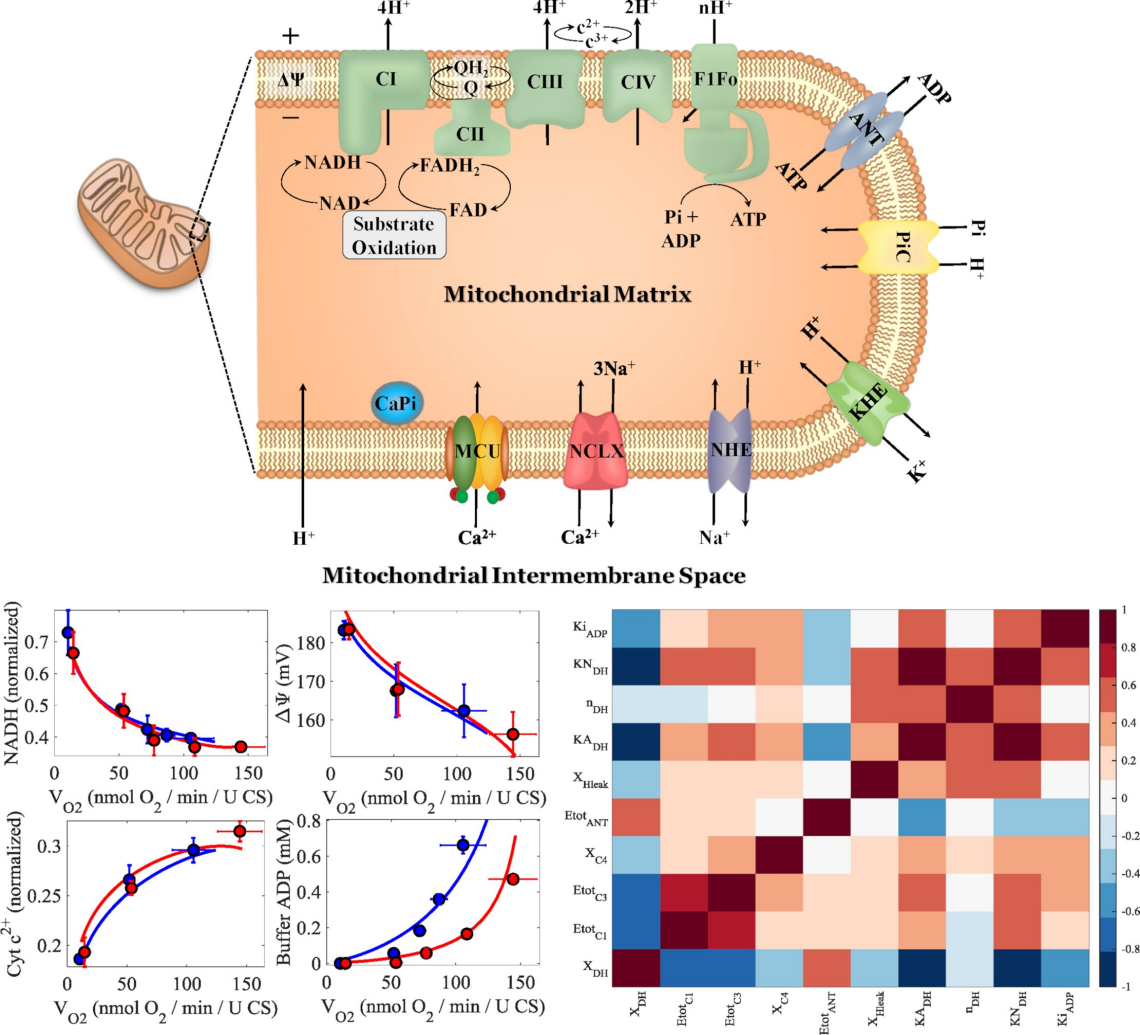
Mitochondrial bioenergetics model diagram, calibration, and parameter correlations. The upper panel shows an updated model diagram of the Bazil et al. model [38] that includes calcium handling reactions. The updated model was fit to the data from Vinnakota et al. [39]. The lower left four panels show model simulations faithfully reproducing the experimental data. The lower right panel shows the parameter correlations represented as a heat map.

Fig 3 shows the updated schematic of the bioenergetics model with new rate expressions and components, the new updated model fits to the experimental data it was originally parameterized on, and the parameter correlations shown as a heat map for the adjustable parameters given in Table 1. As shown in the center four panels in Fig 3, the model fits the data equally well as the original model, and in several cases even better. In particular, the updated model fits are more faithful to the data for the NADH and ADP data. The parameter correlation heatmap shown in the right panel of Fig 3 reveals the model parameters are relatively uncorrelated except for strong correlations between the MitoDH and electron transport related parameters.

**Table 1:** Updated base model parameters.

Parameter	Description	Value	Units	Sensitivity	Rank
X_DH_	Substrate oxidation activity (NADH producing)	248	nmol/mg/min	0.607	1
Etot_C1_	Complex I content	199	pmol/mg	0.178	4
Etot_C3_	Complex III content	417	pmol/mg	0.109	8
X_C4_	Complex IV activity	1.210E+4	nmol/min/mg	0.150	5
Etot_ANT_	Adenine nucleotide translocase content	57.2	nmol/mg	0.019	10
X_Hleak_	Proton leak activity	6.20E+7	nmol/mg/min	0.128	7
KA_DH_	ATPase feedback constant	5.91E-2	M	0.353	2
n_DH_	ATPase feedback Hill coefficient	1.15	-	0.313	3
KN_DH_	NADH/NAD^+^ feedback constant	0.143	M^-1^	0.067	9
Ki_ADP_	K_i_ for ADP of external ATPase flux	262	μM	0.140	6

Local sensitivity coefficients are normalized and averaged with all model outputs aligned with the experimental data. Only non-zero sensitivity coefficients were averaged. The leak activity for the model simulations of the guinea pig data (calcium-dependent inhibition data) was lowered by 40% to account for the tighter respiratory coupling that these mitochondria possess.

There is lot of evidence that calcium is reversibly sequestrated as calcium phosphate granules to maintain energy homeostasis [12, 13]. Although we have implicitly modeled granule formation and calcium storage using an empirical model [46], this approach required an artificially high proton buffering capacity to prevent excess matrix alkalization. This over alkalization was corrected by explicitly modeling calcium phosphate granule formation and including proton release as a part of this process [13]. A general reaction formula for calcium phosphate formation is depicted in Eq 1. This equation is charge and atom balanced and works for many types of calcium phosphate stoichiometries including tricalcium phosphate, hydroxyapatite, and octacalcium phosphate. In the case that 3*m*-2*n* < 0, the H in the calcium phosphate complex is understood to be OH with a stoichiometry of 2*n*-3*m*.

$$nCa^{2+}+mHPO_{4}^{2-}\underset{K_{CaPi}}{↔}Ca_{n}{\left(PO_{4}\right)}_{m}H_{3m-2n}+2(n-m)H^{+}$$ (1)

Assuming an additional prototypical buffering component, the total matrix calcium buffering power that includes the formation of calcium phosphate is shown in Eq 2.

$$\beta_{Ca}=[B_{Ca}]/K_{Ca}/{\left(1+[Ca^{2+}]/K_{Ca}\right)}^{2}+K_{CaPi}{\left[Ca^{2+}\right]}^{n-1}{\left[HPO4^{2-}\right]}^{m}/{\left[H^{+}\right]}^{2m-2n}$$ (2)

In Eq 2, we use the concentration of hydrogen phosphate, as opposed to the phosphate ion, for maximum compatibility and simplicity. The bioenergetics model does not include the phosphate ion as a state variable because this ion only exists in appreciable quantities under very basic, non-physiological conditions. Including the phosphate ion would not change the results but require addition state variables and parameters to be added to the model.

**Fig 4:**
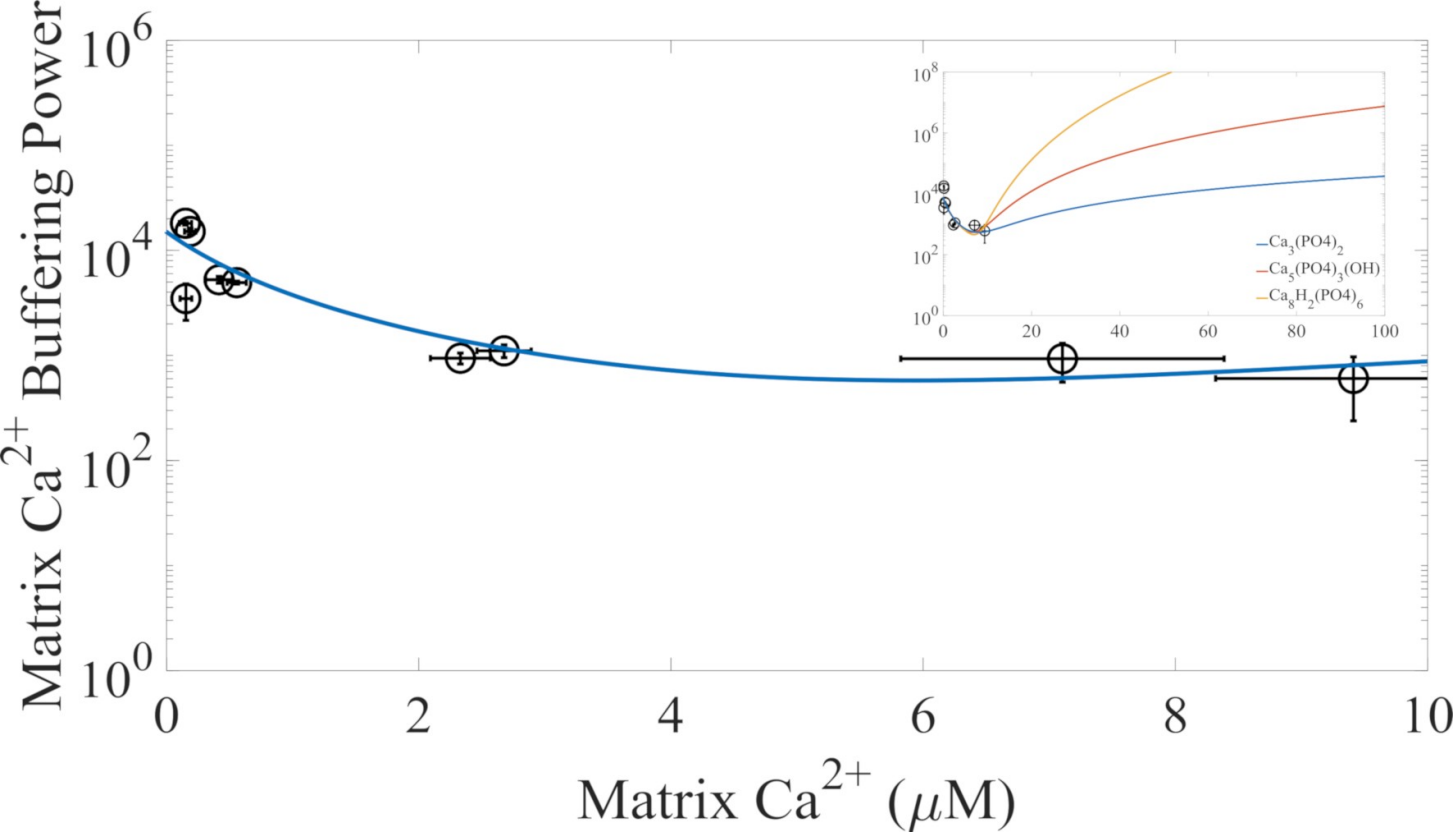
Mitochondrial calcium buffering model. The mitochondrial calcium buffering data from Blomeyer et al. [44] for the conditions most similar to the conditions in this study was used to fit the new calcium phosphate buffering system using an explicit representation of calcium phosphate granule formation. In the inset, the buffering power for the tricalcium phosphate, hydroxyapatite, and octacalcium phosphate are shown for higher matrix calcium concentrations. These simulations were performed with the following matrix conditions: pH 7.4, [K^+^] = 150 mM, [Na^+^] = 5 mM, [Mg^2+^] = 0.5 mM, and [Pi]_tot_ = 10 mM.

Fig 4 shows the relationship between mitochondrial calcium buffering power and matrix free calcium with the explicit representation of calcium phosphate granule formation compared to the experimental data from Blomeyer et al. [44]. The model consists of two essential components. The first component characterizes prototypical calcium buffering caused by binding to proteins and lipids not explicitly accounted for in the model. The second component represents the formation calcium phosphate granules. Table 2 gives the parameters used to simulate the data given in Fig 4. The calcium phosphate complexation constant is not a solubility product constant. It is a parameter that governs the matrix calcium buffering power. For this data, either tricalcium phosphate, hydroxyapatite, or octacalcium phosphate formation fit the data with near equality. As shown in the inset of Fig 4, the major difference between the three types of calcium phosphates is that higher buffering powers for matrix calcium concentrations exceeding 10 μM are obtained as calcium stoichiometric coefficient increases. For simplicity, we chose to model tricalcium phosphate formation. In addition, we assume calcium phosphate precipitate formation is rapid relative to calcium uptake and efflux. Further experiments are required to determine which calcium phosphate species constitute the main precipitate.

**Table 2:** Calcium phosphate precipitate and buffering parameters.

Parameter	Definition	Value	Unit
[*B*_*Ca*_]	Total calcium binding sites	15	mM
*K*_*Ca*_	Ca^2+^ binding constant	2	μM
*K*_*CaPi*_	CaPi complexation constant	50	M^-2^
*n*	Calcium stoichiometry	3	-
*m*	Phosphate stoichiometry	2	-
*k*_*f*_	Arbitrarily high forward rate constant	10^21^	M^-4^ min^-1^

We then proceeded to simultaneously fit the model to the data shown in Fig 1 by allowing some of the calcium related parameters to change. For these simulations, we ignored the calcium-dependent reduction in ADP-stimulated respiration to get a baseline model capable of simulating the calcium handling data. To minimize the number of adjustable parameters, we selected the most important parameters with respect to calcium handling using sensitivity analysis conducted with estimated parameter values obtained from the literature. These parameters are given in Table 3 and were determined to be the MCU activity, MCU calcium dissociation constant, NCLX activity, and the NCLX calcium dissociation constant. All other model parameters used for the simulations are given in the Supporting Information (S1 File). The most sensitive parameter is the MCU activity. This parameter has a high, normalized local sensitivity coefficient relative to the others. The next highest ranked sensitivity is for the MCU calcium dissociation constant. The remaining two parameters, the NCLX activity and the NCLX calcium dissociation constant have near equal sensitivity values. The parameters were relatively correlated (between 0.7 and 1) which indicates additional information is needed to uniquely identify these values. Also, the NCLX parameters are dependent on the matrix calcium buffering parameters given in Table 2. However, these correlations do not impact the model results in this study.

**Table 3:** Adjustable calcium related parameters.

Parameter	Description	Value	Units	Sensitivity	Rank
*X*_*MCU*_	MCU activity	3.96E+4	nmol/mg/min	0.734	1
*K*_*Ca*,*MCU*_	MCU calcium dissociation constant	4.8	mM	0.593	2
*X*_*NCLX*_	NCLX activity	104	nmol/mg/min	0.291	3
*K*_*Ca*,*NCLX*_	NCLX calcium dissociation constant	240	μM	0.255	4

Local sensitivity coefficients are normalized and averaged with all model outputs are aligned with the experimental data. Only non-zero sensitivity coefficients were averaged.

**Fig 5:**
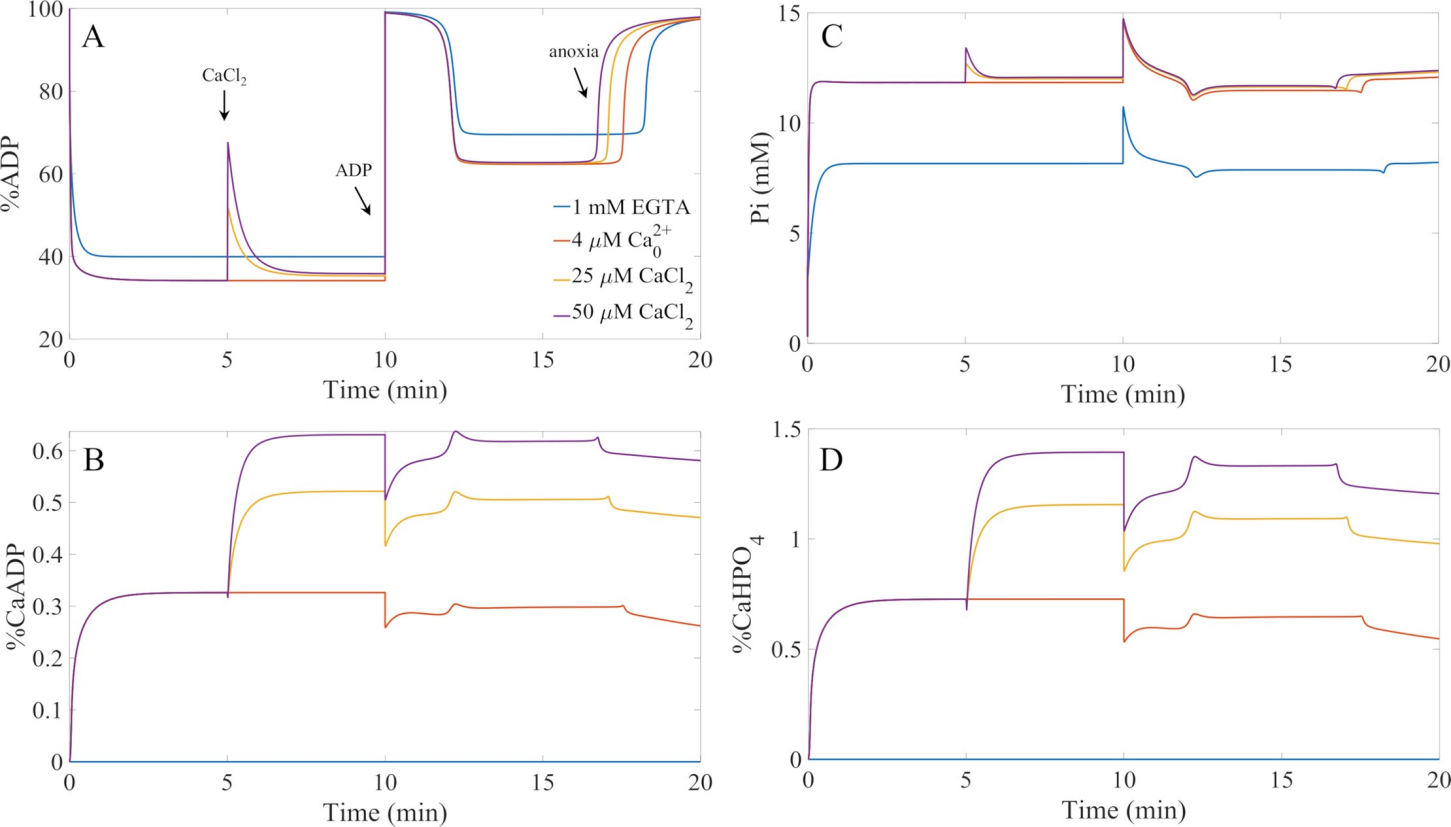
Calcium complexation with ATP synthase substrates does not explain the calcium-induced inhibition of ADP-stimulated respiration. A) Simulations of the percent of matrix ADP during the calcium loading and ADP bolus experiments given in Fig 1. The times when the CaCl_2_ and ADP boluses, as well as, when the environment becomes anoxic are marked. These times are the same for all panels. B) The fraction of matrix ADP chelated with calcium for the same simulation. C) The simulated concentrations of matrix Pi during the experiments. D) The corresponding simulations of solvated calcium phosphate species (not calcium phosphate precipitate) during the experiments. The simulation protocols were as faithful to the experimental protocols as reasonably possible.

With the model calibrated, we began to computationally test the remaining hypotheses. Fig 5 shows that the reduction in ADP-stimulated respiration is not due to a reduction in available ADP and/or phosphate for oxidative phosphorylation. Even the highest calcium bolus only leads to less than 1% and 2% reduction in available ADP and phosphate, respectively. This makes it highly unlikely that this is the cause of the calcium-dependent reduction in respiration shown in Fig 1. However, these simulations do not rule out the possibility that ADP is physically incorporated into the calcium phosphate granules. That remains a possibility to be tested, thus the hypothesis is not fully disproven. We will revisit this hypothesis below.

We next tested the remaining hypothesis by adding a calcium-dependent Michaelis Menten-like inhibitory function to the main reactions involved in ADP-stimulated respiration. We opted to use a simple inhibitory function as opposed to more non-linear Hill type functions for simplicity. For this, we fit the adjustable parameters given in Table 3 to simulate the calcium handling components of the mitochondrial bioenergetics model for the experimental conditions described in Fig 1. All other parameters were fixed and given in detail in the Supporting Information (S1 File). Table 4 summarizes the results and shows that the best mechanism that matches the data is the inhibition of complex I as a function of the concentration calcium phosphate granules. The next best mechanism is the inhibition of complex III as a function of calcium phosphate granule concentration. All other conditions did not lead to adequately good fits to the data based on the normalized least squares values and visual inspection. The relatively small inhibition constants for the inorganic phosphate carrier and F1FO ATP synthase are expected, as these rate expressions have high activities to put them in a near equilibrium state. More complex inhibition functions involving multiple reactions were not considered. These results show that the most likely site of inhibition is at complex I or III and the calcium phosphate granule concentration is the likely cause.

**Table 4:** Matrix free calcium and calcium phosphate inhibition constants.

Inhibited Reaction	K_I_ for Matrix Ca^2+^	Units	Percent change from best fit	K_I_ for CaPi	Units	Percent change from best fit
TCA cycle	88.4	μM	144	294	mM	132
Complex I	49.8	μM	124	163	mM	Best fit
Complex III	6.27	μM	119	20.0	mM	111
Complex IV	6.50	μM	131	22.9	mM	129
Adenine Nucleotide Translocase	3.65	μM	167	9.63	mM	140
Inorganic Phosphate Carrier	1.92	μM	146	2.00	mM	123
F_1_F_O_ ATP Synthase	0.497	μM	211	1.61	mM	182

Fits were quantified using a normalized least squares value which was computed by summing the square of the difference between model and data relative to the data and divided by the total number of data points.

**Fig 6:**
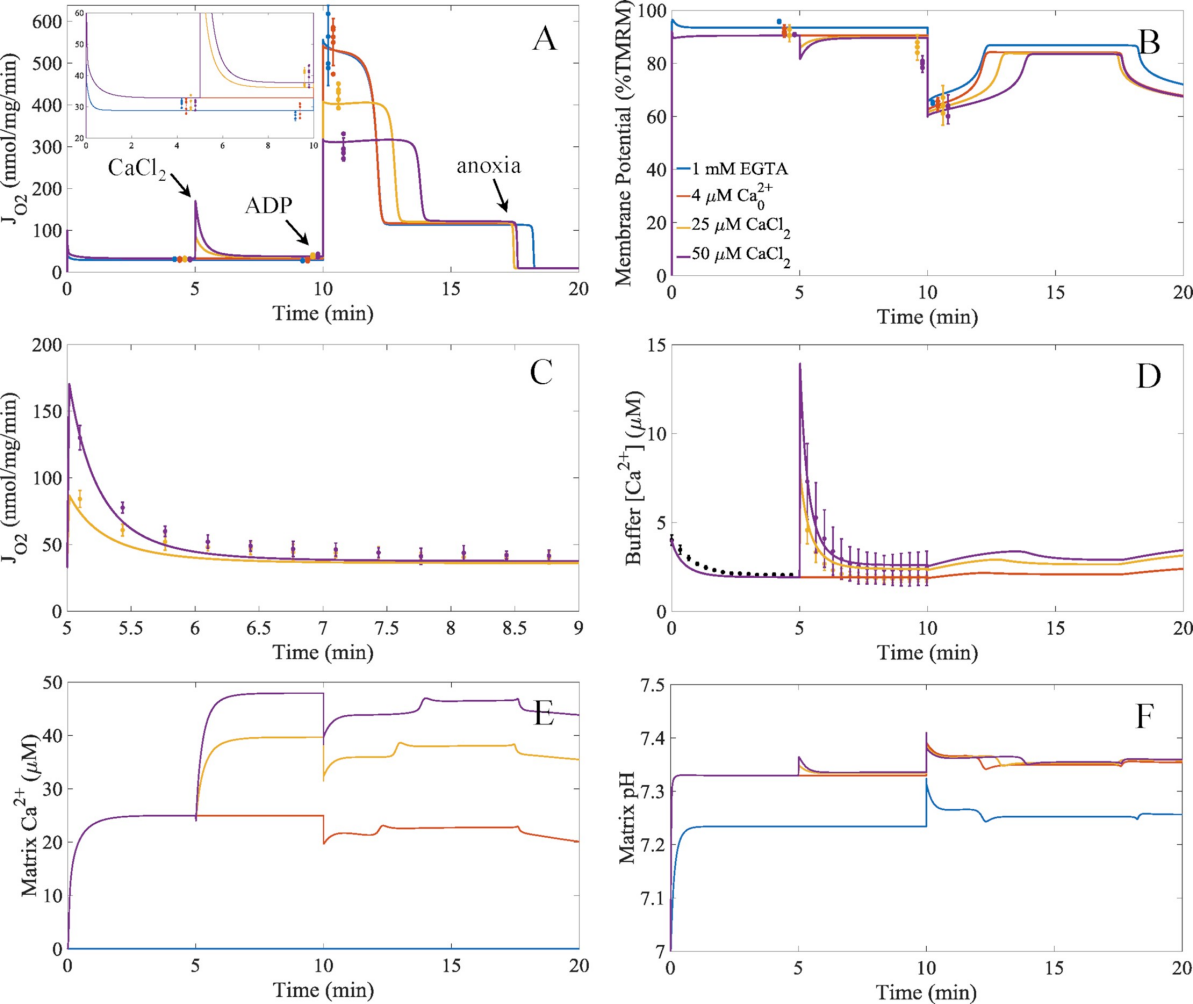
Model simulations compared to the experimental data of the calcium-induced inhibition of ADP-stimulated respiration. A) Model simulations of respiration for the different calcium conditions for the protocol detailed in Fig 1. The inset shows the model simulations for leak and sodium/calcium cycling respiratory states in more detail. B) Model simulations of membrane potential dynamics. The model output for membrane potential was converted to a percent basis from a theoretical 200 mV maximum so that it was comparable to the normalized TMRM data. C) Simulations of the stimulatory effect of calcium uptake on respiration. D) Simulations of the buffer calcium dynamics. E) Simulations of the matrix calcium dynamics. F) Simulations of the matrix pH dynamics. These simulations were carried out assuming that Complex I was inhibited by the concentration of calcium phosphate granules using the inhibitory constant given in Table 4. To remain faithful to the experimental conditions, we added an ATPase reaction that represents the typical ATPase contaminate that is present in all isolated mitochondrial preps. For details, see the Supporting Information (S1 File). Error bars are given as 95% confidence intervals. Individual data are represented as dots. The colors for each panel represent the conditions given in the legend of panel B.

The model results using the complex I inhibition mechanism compared to the experimental data is shown in Fig 6. As shown, the model faithfully reproduces the experimental data. The model captures the calcium-dependent stimulation of respiration caused by sodium/calcium cycling and the inhibitory effect of calcium phosphate precipitates on ADP-stimulated respiration. In addition, the model reveals why the mitochondrial membrane potential is more polarized during leak state respiration when 1 mM EGTA is present. With the model, we can mechanistically explain this observation. The reason why the membrane potential is higher in this respiratory state when 1 mM EGTA is present, the matrix pH is closer to the buffer pH. This lowers the ΔpH component of the proton motive force and leads to an increase in the membrane potential. The matrix alkalization when 1 mM EGTA is not present is due to uptake of the residual calcium in the buffer. This residual calcium contaminate comes from reagent impurities mainly coming from potassium chloride and potassium phosphate salts. This is a small, but important, validation of the model ability to accurately simulate mitochondrial physiology.

### Experimentally testing model driven hypotheses

**Fig 7:**
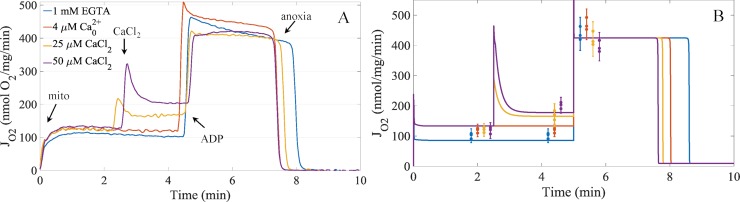
Representative experimental respiratory dynamics and model simulations of the effects of calcium on succinate supported respiration in the presence of rotenone. A) Respiratory dynamics demonstrating the effects calcium on mitochondria energized with succinate with complex I inhibitor rotenone. B) Model simulations of the experiments shown in panel A) with experimental data for leak, sodium/calcium cycling, and APD-stimulated respiratory states overlaid on top of the model simulations. Error bars are given as 95% confidence intervals. Individual data are represented as dots. The colors in panel B represent the same conditions as given by the legend in panel A.

We used the model results given in Table 4 to devise an experiment to further rule out as many of the model-generated hypotheses as possible. Since all the reactions given in Table 4 have some dependence on NADH-dependent reactions, we chose to perform a similar experiment shown in Fig 1 but use succinate as the fuel to bypass any NADH-dependent reactions. The results from those experiments are shown in Fig 7A. To simulate the experiments, the two parameters from the empirical rate expression in Bazil et al. [38] for succinate-dependent respiration was modified to match the leak state and ADP-stimulated respiratory rates when 1 mM EGTA was present. For details, see the Supporting Information (S1 File). While the calcium boluses result in a minor decrease in ADP-stimulated respiration, the magnitude is far less than that of ADP-stimulated respiration using NADH-linked respiratory fuels. As can be seen, the model faithfully reproduces the experimental data supporting the hypothesis that calcium phosphate granules lead to complex I inhibition which lowers the ADP-stimulated respiration rates in a titratable manner. These results dispute the notion that ADP is sequestered in these calcium phosphate granules because the ADP-stimulated respiration rates are hardly reduced. These results also rule out the complex III inhibition mechanism as the primary mechanism explaining the calcium-dependent inhibition of ADP-stimulated respiration, and only leaves calcium phosphate dependent inhibition of complex I.

## Discussion

Ischemic myocardium is characterized by a chronic elevation in cellular calcium concentration. In this setting, mitochondria accumulate calcium to levels that compromise their ability to synthesize ATP. The calcium accumulated during ischemia is mostly present in the form of insoluble calcium phosphate granules [12–15]. Our experimental results show that calcium levels below thresholds that trigger mitochondrial permeability transition significantly lowers the rate of ADP-stimulated respiration. Some studies from rat heart, liver and brain mitochondria suggest that matrix calcium overload can directly inhibit ANT activity and reduce the ATP synthase rate [28, 29, 47]. Other studies demonstrate that the flux of pyruvate dehydrogenase complex can also be inhibited with matrix calcium overload [23]. In addition to these studies, there are reports that calcium stimulates cytochrome c dissociation from the membrane and alters complex III and IV activities [48]. Still others link calcium overload and complex I activity so that in the face of elevated levels of calcium, the ATP synthase rate is significantly reduced [20, 22]. Calcium-dependent matrix proteases have also been implicated in calcium-induced mitochondria dysfunction [33, 34]. These calcium effects on mitochondrial metabolism are not necessary mutually exclusive. There could be a spectrum of calcium overload responses that depend on tissue, environment, and extent of ischemia/reperfusion injury.

To understand the underlying mechanism of calcium inhibition on ADP-induced respiration, we combined both experimental and computational approaches using the classic model-based design of experiments paradigm. The experimental approach utilized the use of isolated mitochondria and interrogated the respiratory, energetic, and calcium handling response to various boluses of calcium and mimic the calcium overload conditions that occur during ischemia. These responses were quantified and used to inform a computational model capable of explaining the calcium-dependent inhibition of ADP-stimulated respiration. The model used in this study includes mechanistic descriptions of mitochondrial metabolism and energetics in addition to calcium uptake, sequestration, and efflux pathways. In doing so, the first set of experiments produced a list of hypotheses which we began to systematically rule out until left with one possible explanation of the data.

The results show that oxidative phosphorylation inhibition by calcium overload is due to the formation of calcium phosphate precipitation, not matrix free calcium, inhibiting complex I. This hypothesis is further supported by experimental data showing no inhibition of isolated complex I activity by calcium [21]. Electron micrograph images have localized these phosphate granules in the matrix near or attached to the inner membrane [15, 49]. A possible explanation of these findings is that calcium phosphate granules act as physical barriers or disrupt complex I activity. Perhaps, complex I is at or near the nucleation site for granule formation and growth. Alternatively, calcium phosphate granules may disrupt cristae structure and impair mitochondrial function. We found no reports suggesting these mechanisms, so they are speculative and remain to be tested.

Our isolated mitochondrial results are supported by several *in situ* findings. For example, mitochondria isolated from whole hearts subjected to ischemia/reperfusion (IR) injury contain large quantities of calcium and depressed ATP synthesis rates [50–53]. In addition, a major target of IR injury is complex I inhibition [54–56]. These findings are in direct support of our isolated mitochondrial work. Moreover, mitochondrial calcium content in excess of 10 nmol/mg protein results in the formation of calcium phosphate granules [57]. In our study, mitochondria were loaded with calcium content in the range of 0–500 nmol calcium/mg protein. Thus, our experimental results with isolated mitochondria capture the major features associated with IR injury where complex I activity is inhibited in response to calcium accumulation and calcium phosphate precipitation prior to the onset mitochondrial permeability transition.

The dynamic model of mitochondrial bioenergetics developed in this study served as a powerful tool to help analyze the experimental data. With only five adjustable parameters (two MCU, two NCLX, and a calcium inhibition constant), the model is capable of simulating experimental results from two types of respiratory substrates each with four different calcium conditions. And while the model simulations do not pass through all the data points, they faithfully recapitulate the physiological data in a self-consistent, thermodynamically balanced manner. More complex rate expressions for the MCU and NCLX or the inclusion of the sodium-independent calcium efflux pathway [58, 59] may increase the quantitative agreement between the model and experimental data; however, this would require many more additional parameters and would only marginally improve the model fits. Regardless, the underlying mechanistic information provided by the model would not change.

In summary, we show that mitochondrial respiration with complex I substrates during oxidative phosphorylation is inhibited by calcium phosphate accumulation. Respiration with complex II substrates is nearly independent of calcium phosphate accumulation and only shows a very modest decrease in respiration. This decrease in respiration can be attributed to calcium phosphate granules interfering with additional enzymes in addition to complex I. Further experimental work is needed to verify these findings. For example, direct imagining of calcium phosphate granules and their effect on cristae structure using electron cryomicroscopy with coincident mitochondrial functional data could finally elucidate the mechanism behind calcium overload and oxidative phosphorylation inhibition.

## Methods

### Ethics statement

The work presented herein conformed to the National Institutes of Health’s Guild for the Care and Use of Laboratory Animals and was approved by Michigan State University’s Institutional Animal Care and Use Committee. Hartley guinea pigs (4–6 weeks) were anesthetized with 5% isoflurane. Euthanasia was carried out by decapitation.

### Experimental methods

All chemicals and reagents were purchased from Sigma Aldrich unless otherwise stated. Calcium Green 5N was purchased from Molecular Probes. Cardiac mitochondria were isolated from guinea pigs (350–450 g) using differential centrifugation as described in Wollenman et al. [60]. Briefly, isolated mitochondria were suspended at 40 mg/ml in isolation buffer consisting of 200 mM mannitol, 50 mM sucrose, 5 mM dibasic potassium phosphate, 5 mM MOPS, 1 mM EGTA, and 0.1% (w/v) BSA at pH 7.15. Protein was quantified using the BCA assay and BSA standards. To test the effect of calcium on respiration and membrane potential, 0.1 mg/ml mitochondria were suspended in respiration buffer consisting of 130 mM potassium chloride, 5 mM dibasic potassium phosphate, 1 mM magnesium chloride, 20 mM MOPS, 0.1% (w/v) BSA at pH 7.1 at 37°C. Due to chemical impurities, the free calcium concentration in the absence of EGTA in the respiration buffer was 4.01 +/- 0.43 μM. Sodium pyruvate (5 mM) and L-malate (1 mM) or disodium succinate (10 mM) and complex I inhibitor rotenone (0.5 μM) were used to energize the mitochondria. ADP was added as a bolus at a concentration of 500 μM. Respiration was measured using an Oroboros O2k, and membrane potential was measured using an Olis DM245 spectrofluorometer and 0.1 μM of the lipophilic cationic dye, TMRM. The ratiometric approach (546/573 nm excitation, 590 nm emission) by Scaduto et al. [61] was used. TMRM fluorescent data were normalized to the maximum value obtained using alamethicin (25 μg/ml) and the protonophore, carbonyl cyanide m-chlorophenyl hydrazine (1 μM), and the minimum value obtained during the leak respiratory state using oligomycin (1 μM) and nigericin (2 μM). The signal was then transformed using the following equation: *%TMRM*(*t*) = 100*(*R*_*max*_−*R*(*t*))/(*R*_*max*_-*R*_*min*_). Buffer calcium contamination was measured using 1 μM of the fluorescent indicator calcium green 5N (503 nm excitation, 532 nm emission). The measured K_D_ in our conditions was 30 μM obtained by titrating free calcium concentrations using total CaCl_2_ and EGTA concentrations given by the MaxChelator program [62].

The experimental protocol was as follows: at 0 minutes mitochondria plus 5mM sodium pyruvate and 1 mM L-malate were added; at 5 minutes, a bolus of CaCl_2_ (0 μM, 25 μM and 50 μM) was given; at 10 minutes, 500 μM of ADP was added; the respiratory rate was followed for an additional 10 to 20 min. When EGTA was present, no calcium bolus was added. The protocol for measuring membrane potential using TMRM was identical, except that the measurements were carried out in a cuvette open to atmosphere. In other respiratory trials, 10 mM disodium succinate and 0.5 μM rotenone was added with the mitochondria. In these trials, the boluses of CaCl_2_ were added at 2.5 mins. The 500 μM ADP bolus was added at 5 min.

### Calpain inhibitors

Calpain I, II, and III inhibitors and the calpeptin inhibitor were purchased from Sigma. The calpain 10 inhibitor was synthesized using the Sigma custom peptide synthesis service. The peptide sequence (CYGAK) was identical to the sequence reported in Rasbach et al. [33]. Calpain inhibitors (10 μM) were incubated in the presence of energized mitochondria for five minutes before the addition of the CaCl_2_ boluses. Five minutes after the CaCl_2_ boluses, a 500 μM bolus of ADP was added.

### Statistical analysis

Data are presented as mean +/- standard deviation or 95% confidence intervals, as noted. Data were analyzed and plotted using MATLAB. Confidence intervals were computed using t and chi-square distributions for uncensored samples using an exact method. Data were checked and confirmed for normality using the Shapiro-Wilk test. Statistical significance was tested using an unbalanced ANOVA followed by a post-hoc Tukey’s range test. The *n* values ranged from 3 to 20 for each condition tested.

### Computational model

The computational model of cardiac mitochondrial energetics from Bazil et al. [38] was used as a framework and updated to include additional reactions and rate equations for sodium and calcium homeostasis. A new lumped TCA cycle flux expression was developed, and a new model of complex III kinetics [45] was incorporated into the mitochondrial bioenergetics model. Some sodium and calcium flux expressions and dissociation constants from Bazil et al. [40] or Bazil et al. [46] were also added to the model. Other minor changes were made to the model and discussed further in the Results and Discussion section and given in the Supporting Information (S1 File). Model codes are available in the Supporting Information (S2 File).

The DAE’s of the model were numerically integrated using MATLAB (R2017b) and the stiff ode solver ode15s using a relative error tolerance of 10^−4^ and an absolute error tolerance and 10^−9^. The implicit method using backward differentiation formulas was used to solve the system of DAEs. Parameter optimization was done using a desktop PC (64-bit operating system and x64-based processor Intel® core ™ i7-7700 CPU @3.60GHz and 16 GB RAM) using the Parallel Computing Toolbox. Manual determination of parameter upper and lower bounds was followed by the gradient-based optimization algorithm, fmincon.

## Supporting information

S1 File This is the supporting information containing a detailed description of the computer model used in this study. (DOCX)

S2 File This is a zip of the MATLAB computer codes of the model used in this study. (ZIP)

## References

[1] Stanley WC. Myocardial energy metabolism during ischemia and the mechanisms of metabolic therapies. J Cardiovasc Pharmacol Ther. 2004;9 Suppl 1:S31–45. 10.1177/107424840400900104 . 15378130

[2] Andrienko TN, Picht E, Bers DM. Mitochondrial free calcium regulation during sarcoplasmic reticulum calcium release in rat cardiac myocytes. J Mol Cell Cardiol. 2009;46(6):1027–36. 10.1016/j.yjmcc.2009.03.015 19345225 PMC2683203

[3] Gunter TE, Pfeiffer DR. Mechanisms by which mitochondria transport calcium. Am J Physiol. 1990;258(5 Pt 1):C755–86. 10.1152/ajpcell.1990.258.5.C755 . 2185657

[4] Baughman JM, Perocchi F, Girgis HS, Plovanich M, Belcher-Timme CA, Sancak Y, et al Integrative genomics identifies MCU as an essential component of the mitochondrial calcium uniporter. Nature. 2011;476(7360):341–5. 10.1038/nature10234 21685886 PMC3486726

[5] McCormack JG, Halestrap AP, Denton RM. Role of calcium ions in regulation of mammalian intramitochondrial metabolism. Physiol Rev. 1990;70(2):391–425. 10.1152/physrev.1990.70.2.391 . 2157230

[6] McCormack JG, Denton RM. Influence of calcium ions on mammalian intramitochondrial dehydrogenases. Methods Enzymol. 1989;174:95–118. . 2561175 10.1016/0076-6879(89)74013-1

[7] Griffiths EJ, Rutter GA. Mitochondrial calcium as a key regulator of mitochondrial ATP production in mammalian cells. Biochim Biophys Acta. 2009;1787(11):1324–33. 10.1016/j.bbabio.2009.01.019 . 19366607

[8] Luongo TS, Lambert JP, Gross P, Nwokedi M, Lombardi AA, Shanmughapriya S, et al The mitochondrial Na(+)/Ca(2+) exchanger is essential for Ca(2+) homeostasis and viability. Nature. 2017;545(7652):93–7. 10.1038/nature22082 28445457 PMC5731245

[9] Palty R, Silverman WF, Hershfinkel M, Caporale T, Sensi SL, Parnis J, et al NCLX is an essential component of mitochondrial Na+/Ca2+ exchange. Proc Natl Acad Sci U S A. 2010;107(1):436–41. 10.1073/pnas.0908099107 20018762 PMC2806722

[10] Steenbergen C, Murphy E, Levy L, London RE. Elevation in cytosolic free calcium concentration early in myocardial ischemia in perfused rat heart. Circ Res. 1987;60(5):700–7. . 3109761 10.1161/01.res.60.5.700

[11] Miyata H, Silverman HS, Sollott SJ, Lakatta EG, Stern MD, Hansford RG. Measurement of mitochondrial free Ca2+ concentration in living single rat cardiac myocytes. Am J Physiol. 1991;261(4 Pt 2):H1123–34. 10.1152/ajpheart.1991.261.4.H1123 . 1928394

[12] Lehninger AL. Mitochondria and calcium ion transport. Biochem J. 1970;119(2):129–38. 4922961 10.1042/bj1190129 PMC1179333

[13] Chalmers S, Nicholls DG. The relationship between free and total calcium concentrations in the matrix of liver and brain mitochondria. J Biol Chem. 2003;278(21):19062–70. 10.1074/jbc.M212661200 . 12660243

[14] Chinopoulos C, Adam-Vizi V. Mitochondrial Ca2+ sequestration and precipitation revisited. FEBS J. 2010;277(18):3637–51. 10.1111/j.1742-4658.2010.07755.x . 20659160

[15] Kristian T, Pivovarova NB, Fiskum G, Andrews SB. Calcium-induced precipitate formation in brain mitochondria: composition, calcium capacity, and retention. J Neurochem. 2007;102(4):1346–56. 10.1111/j.1471-4159.2007.04626.x 17663756 PMC2566803

[16] Ferrari R. Myocardial Ischemia and Lipid Metabolism: Springer US; 2012.

[17] Griffiths EJ, Halestrap AP. Mitochondrial non-specific pores remain closed during cardiac ischaemia, but open upon reperfusion. Biochem J. 1995;307 (Pt 1):93–8. 7717999 10.1042/bj3070093 PMC1136749

[18] Bernardi P, Di Lisa F. The mitochondrial permeability transition pore: molecular nature and role as a target in cardioprotection. J Mol Cell Cardiol. 2015;78:100–6. 10.1016/j.yjmcc.2014.09.023 25268651 PMC4294587

[19] Chouchani ET, Pell VR, Gaude E, Aksentijevic D, Sundier SY, Robb EL, et al Ischaemic accumulation of succinate controls reperfusion injury through mitochondrial ROS. Nature. 2014;515(7527):431–5. 10.1038/nature13909 25383517 PMC4255242

[20] Brustovetsky N, Brustovetsky T, Jemmerson R, Dubinsky JM. Calcium-induced cytochrome c release from CNS mitochondria is associated with the permeability transition and rupture of the outer membrane. J Neurochem. 2002;80(2):207–18. . 11902111 10.1046/j.0022-3042.2001.00671.x

[21] Pandya JD, Nukala VN, Sullivan PG. Concentration dependent effect of calcium on brain mitochondrial bioenergetics and oxidative stress parameters. Front Neuroenergetics. 2013;5:10 10.3389/fnene.2013.00010 24385963 PMC3866544

[22] Hardy L, Clark JB, Darley-Usmar VM, Smith DR, Stone D. Reoxygenation-dependent decrease in mitochondrial NADH:CoQ reductase (Complex I) activity in the hypoxic/reoxygenated rat heart. Biochem J. 1991;274 (Pt 1):133–7. 1900416 10.1042/bj2740133 PMC1149930

[23] Lai JC, DiLorenzo JC, Sheu KF. Pyruvate dehydrogenase complex is inhibited in calcium-loaded cerebrocortical mitochondria. Neurochem Res. 1988;13(11):1043–8. . 3237304 10.1007/BF00973148

[24] Lai JC, Cooper AJ. Brain alpha-ketoglutarate dehydrogenase complex: kinetic properties, regional distribution, and effects of inhibitors. J Neurochem. 1986;47(5):1376–86. . 3760866 10.1111/j.1471-4159.1986.tb00768.x

[25] Bernard PA, Cockrell RS. Calcium transport by rat brain mitochondria and oxidation of 2-oxoglutarate. Biochim Biophys Acta. 1984;766(3):549–53. . 6548153 10.1016/0005-2728(84)90113-0

[26] Thorne RF, Bygrave FL. Inhibition by calcium of adenine nucleotide translocation in mitochondria isolated from Ehrlich ascites tumour cells. FEBS Lett. 1974;41(1):118–21. . 4852463 10.1016/0014-5793(74)80968-3

[27] Fagian MM, da Silva LP, Vercesi AE. Inhibition of oxidative phosphorylation by Ca2+ or Sr2+: a competition with Mg2+ for the formation of adenine nucleotide complexes. Biochim Biophys Acta. 1986;852(2–3):262–8. . 3022807 10.1016/0005-2728(86)90231-8

[28] Roman I, Clark A, Swanson PD. The interaction of calcium transport and ADP phosphorylation in brain mitochondria. Membr Biochem. 1981;4(1):1–9. . 7219193 10.3109/09687688109065419

[29] Moreno-Sanchez R. Inhibition of oxidative phosphorylation by a Ca2+-induced diminution of the adenine nucleotide translocator. Biochim Biophys Acta. 1983;724(2):278–85. . 6309222 10.1016/0005-2728(83)90146-9

[30] Fink BD, Bai F, Yu L, Sivitz WI. Regulation of ATP production: dependence on calcium concentration and respiratory state. Am J Physiol Cell Physiol. 2017;313(2):C146–C53. 10.1152/ajpcell.00086.2017 . 28515085

[31] Vygodina TV, Mukhaleva E, Azarkina NV, Konstantinov AA. Cytochrome c oxidase inhibition by calcium at physiological ionic composition of the medium: Implications for physiological significance of the effect. Biochim Biophys Acta. 2017;1858(12):982–90. 10.1016/j.bbabio.2017.08.011 . 28866381

[32] Abou-Khalil S, Abou-Khalil WH, Yunis AA. Inhibition of Ca2+ of oxidative phosphorylation in myeloid tumor mitochondria. Arch Biochem Biophys. 1981;209(2):460–4. . 6945822 10.1016/0003-9861(81)90303-9

[33] Rasbach KA, Arrington DD, Odejinmi S, Giguere C, Beeson CC, Schnellmann RG. Identification and optimization of a novel inhibitor of mitochondrial calpain 10. J Med Chem. 2009;52(1):181–8. 10.1021/jm800735d 19072163 PMC2801808

[34] Thompson J, Hu Y, Lesnefsky EJ, Chen Q. Activation of mitochondrial calpain and increased cardiac injury: beyond AIF release. Am J Physiol Heart Circ Physiol. 2016;310(3):H376–84. 10.1152/ajpheart.00748.2015 26637561 PMC4796621

[35] Petronilli V, Cola C, Massari S, Colonna R, Bernardi P. Physiological effectors modify voltage sensing by the cyclosporin A-sensitive permeability transition pore of mitochondria. J Biol Chem. 1993;268(29):21939–45. . 8408050

[36] Goll DE, Thompson VF, Li H, Wei W, Cong J. The calpain system. Physiol Rev. 2003;83(3):731–801. 10.1152/physrev.00029.2002 . 12843408

[37] Smith MA, Schnellmann RG. Calpains, mitochondria, and apoptosis. Cardiovasc Res. 2012;96(1):32–7. 10.1093/cvr/cvs163 22581845 PMC3444233

[38] Bazil JN, Beard DA, Vinnakota KC. Catalytic Coupling of Oxidative Phosphorylation, ATP Demand, and Reactive Oxygen Species Generation. Biophys J. 2016;110(4):962–71. 10.1016/j.bpj.2015.09.036 26910433 PMC4776027

[39] Vinnakota KC, Bazil JN, Van den Bergh F, Wiseman RW, Beard DA. Feedback Regulation and Time Hierarchy of Oxidative Phosphorylation in Cardiac Mitochondria. Biophys J. 2016;110(4):972–80. 10.1016/j.bpj.2016.01.003 26910434 PMC4776028

[40] Bazil JN, Buzzard GT, Rundell AE. A bioenergetic model of the mitochondrial population undergoing permeability transition. J Theor Biol. 2010;265(4):672–90. 10.1016/j.jtbi.2010.06.001 . 20538008

[41] Pradhan RK, Qi F, Beard DA, Dash RK. Characterization of Mg2+ inhibition of mitochondrial Ca2+ uptake by a mechanistic model of mitochondrial Ca2+ uniporter. Biophys J. 2011;101(9):2071–81. 10.1016/j.bpj.2011.09.029 22067144 PMC3207172

[42] Pradhan RK, Beard DA, Dash RK. A biophysically based mathematical model for the kinetics of mitochondrial Na+-Ca2+ antiporter. Biophys J. 2010;98(2):218–30. 10.1016/j.bpj.2009.10.005 20338843 PMC2808480

[43] Kirichok Y, Krapivinsky G, Clapham DE. The mitochondrial calcium uniporter is a highly selective ion channel. Nature. 2004;427(6972):360–4. 10.1038/nature02246 . 14737170

[44] Blomeyer CA, Bazil JN, Stowe DF, Dash RK, Camara AK. Mg(2+) differentially regulates two modes of mitochondrial Ca(2+) uptake in isolated cardiac mitochondria: implications for mitochondrial Ca(2+) sequestration. J Bioenerg Biomembr. 2016;48(3):175–88. 10.1007/s10863-016-9644-1 26815005 PMC5098337

[45] Bazil JN. Analysis of a Functional Dimer Model of Ubiquinol Cytochrome c Oxidoreductase. Biophys J. 2017;113(7):1599–612. 10.1016/j.bpj.2017.08.018 28978450 PMC5627346

[46] Bazil JN, Blomeyer CA, Pradhan RK, Camara AK, Dash RK. Modeling the calcium sequestration system in isolated guinea pig cardiac mitochondria. J Bioenerg Biomembr. 2013;45(3):177–88. 10.1007/s10863-012-9488-2 23180139 PMC3615037

[47] Gomez-Puyou A, Tuena de Gomez-Puyou M, Klapp M, Carafoli E. The effect of calcium on the translocation of adenine nucleotides in rat liver mitochondria. Arch Biochem Biophys. 1979;194(2):399–404. . 443811 10.1016/0003-9861(79)90633-7

[48] Peng TI, Jou MJ. Oxidative stress caused by mitochondrial calcium overload. Ann N Y Acad Sci. 2010;1201:183–8. 10.1111/j.1749-6632.2010.05634.x . 20649555

[49] Greenawalt JW, Rossi CS, Lehninger AL. Effect of Active Accumulation of Calcium and Phosphate Ions on the Structure of Rat Liver Mitochondria. J Cell Biol. 1964;23:21–38. 14228516 10.1083/jcb.23.1.21 PMC2106507

[50] Ferrari R, Pedersini P, Bongrazio M, Gaia G, Bernocchi P, Di Lisa F, et al Mitochondrial energy production and cation control in myocardial ischaemia and reperfusion. Basic Res Cardiol. 1993;88(5):495–512. . 8117254 10.1007/BF00795415

[51] Ferrari R, di Lisa F, Raddino R, Visioli O. The effects of ruthenium red on mitochondrial function during post-ischaemic reperfusion. J Mol Cell Cardiol. 1982;14(12):737–40. . 6187931 10.1016/0022-2828(82)90186-9

[52] Steenbergen C, Fralix TA, Murphy E. Role of increased cytosolic free calcium concentration in myocardial ischemic injury. Basic Res Cardiol. 1993;88(5):456–70. . 8117251 10.1007/BF00795412

[53] Jennings RB, Ganote CE. Structural changes in myocardium during acute ischemia. Circ Res. 1974;35 Suppl 3:156–72. . 4607107

[54] Cairns CB, Ferroggiaro AA, Walther JM, Harken AH, Banerjee A. Postischemic administration of succinate reverses the impairment of oxidative phosphorylation after cardiac ischemia and reperfusion injury. Circulation. 1997;96(9 Suppl):II-260-5. . 9386108

[55] Gadicherla AK, Stowe DF, Antholine WE, Yang M, Camara AK. Damage to mitochondrial complex I during cardiac ischemia reperfusion injury is reduced indirectly by anti-anginal drug ranolazine. Biochim Biophys Acta. 2012;1817(3):419–29. 10.1016/j.bbabio.2011.11.021 22178605 PMC3269517

[56] Sadek HA, Humphries KM, Szweda PA, Szweda LI. Selective inactivation of redox-sensitive mitochondrial enzymes during cardiac reperfusion. Arch Biochem Biophys. 2002;406(2):222–8. . 12361710 10.1016/s0003-9861(02)00446-0

[57] Nicholls DG. Mitochondrial calcium function and dysfunction in the central nervous system. Biochim Biophys Acta. 2009;1787(11):1416–24. 10.1016/j.bbabio.2009.03.010 19298790 PMC2752662

[58] Wingrove DE, Gunter TE. Kinetics of mitochondrial calcium transport. I. Characteristics of the sodium-independent calcium efflux mechanism of liver mitochondria. J Biol Chem. 1986;261(32):15159–65. . 3771569

[59] Crompton M, Heid I, Baschera C, Carafoli E. The resolution of calcium fluxes in heart and liver mitochondria using the lanthanide series. FEBS Lett. 1979;104(2):352–4. . 477998 10.1016/0014-5793(79)80850-9

[60] Wollenman LC, Vander Ploeg MR, Miller ML, Zhang Y, Bazil JN. The effect of respiration buffer composition on mitochondrial metabolism and function. PLoS One. 2017;12(11):e0187523 10.1371/journal.pone.0187523 29091971 PMC5665555

[61] Scaduto RC Jr., Grotyohann LW. Measurement of mitochondrial membrane potential using fluorescent rhodamine derivatives. Biophys J. 1999;76(1 Pt 1):469–77. 10.1016/S0006-3495(99)77214-0 9876159 PMC1302536

[62] Bers DM, Patton CW, Nuccitelli R. A practical guide to the preparation of Ca2+ buffers. Methods Cell Biol. 1994;40:3–29. . 8201981 10.1016/s0091-679x(08)61108-5

