# Supplementary material for: Calcium phosphate precipitation inhibits mitochondrial energy metabolism
Source: PLoS Comput Biol. 2019 Jan 7;15(1):e1006719. doi: 10.1371/journal.pcbi.1006719 (PMC6336351; doi:10.1371/journal.pcbi.1006719)
Supplement: S1 File — (DOCX) [file pcbi.1006719.s001.docx]

**Supporting Information for**

**"Calcium Phosphate Precipitation Inhibits Mitochondrial Energy Metabolism"**

Sathyavani Malyala, Jasiel O. Strubbe, Yizhu Zhang, and Jason N. Bazil

Department of Physiology, Michigan State University, East Lansing, MI 48824

To whom correspondence should be addressed: Jason N. Bazil, Department of Physiology, 567 Wilson Rd., East Lansing, MI, USA, Tel: (517) 884-5124; E-mail: jnbazil@msu.edu

The Supporting Information presents a detailed description of the integrated model used to simulate the results in the main paper. For convenience, the model code is included as supplemental material.

**Integrated Model of Mitochondrial Bioenergetics and Calcium Handling**

The present model is extended from the previous model of mitochondrial bioenergetics[*^1^*](#_ENREF_1). Several flux expressions and model parameters were updated based on the recent works of Bazil et al.[*^2-4^*](#_ENREF_2), as laid out in this model supplemental material section. This supplement consists of four parts. Part 1 lists all the state variables comprising the model, dissociation constants, and physiochemical and general model parameters. Part 2 introduces the set of fifteen non-linear ODEs, four algebraic conservation expressions (for mitochondrial ATP, NADH, UQH_2_, and inter-membrane space c^2+^), and six non-linear cation ODEs (mitochondrial and extra-mitochondrial H^+^, K^+^, Mg^2+^ and Ca^2+^). Part 3 presents the model rate equations and the associated parameter definitions and values. Part 4 describes the code used to simulate the model and generate the plots given in the paper.

The outer-mitochondrial membrane (OMM) is highly permeable to ions, metabolites and substrates of low molecular weight under the given conditions. As such, all permeable inter-membrane space (IMS) state variables were replaced with their respective extra-mitochondrial counterparts, and thus not included in the model. This has the benefit of making the system of non-linear ODEs less stiff, contain fewer state variables, and shortens simulation time without significantly altering the simulation results. The only IMS state variables explicitly simulated in the model are oxidized and reduced forms of cytochrome c (c^3+^ and c^2+^).

**S1 - Model State Variables, Dissociation Constants, and Physiochemical and General Parameter Values**

The state variables used in the model, their definitions, and their units are provided in Table S1.1. All the dissociation constants used in the model are presented in Table S1.2. Dissociation constants are corrected for appropriate temperature (T = 37 ^o^C) and ionic strength (I = 0.17 M), corresponding to the experimental conditions. The physiochemical and general model parameters are presented in Table S1.3.

**Table S1.1.** Model State Variables

| State Variable | Definition | Units |
| --- | --- | --- |
| ΔΨ | Mitochondrial membrane potential | mV |
| *Mitochondrial State Variables* | | |
| [H^+^]_x_ | Mitochondrial free proton concentration | M |
| [Na^+^]_x_ | Mitochondrial free sodium concentration | M |
| [K^+^]_x_ | Mitochondrial free potassium concentration | M |
| [Mg^2+^]_x_ | Mitochondrial free magnesium concentration | M |
| [Ca^2+^]_x_ | Mitochondrial free calcium concentration | M |
| [ATP]_x_ | Total mitochondrial ATP concentration | M |
| [ADP]_x_ | Total mitochondrial ADP concentration | M |
| [Pi]_x_ | Total mitochondrial Pi concentration | M |
| [NADH]_x_ | Total mitochondrial NADH concentration | M |
| [NAD]_x_ | Total mitochondrial NAD concentration | M |
| [UQH_2_]_x_ | Total mitochondrial ubiquinol concentration | M |
| [UQ]_x_ | Total mitochondrial ubiquinone concentration | M |
| [O_2_^.-^]_x_ | Total mitochondrial superoxide concentration | M |
| [H_2_O_2_]_x_ | Total mitochondrial hydrogen peroxide concentration | M |
| [CaPi]_x_ | Calcium phosphate precipitate content | M |
| *Intermembrane Space (IMS) State Variables* | | |
| [c^2+^]_i_ | Total IMS cytochrome c^2+^ (reduced) concentration | M |
| [c^3+^]_i_ | Total IMS cytochrome c^3+^ (oxidized) concentration | M |
| *Extra-Mitochondrial State Variables* | | |
| [H^+^]_e_ | Extra-mitochondrial free proton concentration | M |
| [Na^+^]_e_ | Extra-mitochondrial free sodium concentration | M |
| [K^+^]_e_ | Extra-mitochondrial free potassium concentration | M |
| [Mg^2+^]_e_ | Extra-mitochondrial free magnesium concentration | M |
| [Ca^2+^]_e_ | Extra-mitochondrial free calcium concentration | M |
| [ATP]_e_ | Total extra-mitochondrial ATP concentration | M |
| [ADP]_e_ | Total extra-mitochondrial ADP concentration | M |
| [Pi]_e_ | Total extra-mitochondrial Pi concentration | M |
| [O_2_^.-^]_e_ | Total extra-mitochondrial superoxide concentration | M |
| [H_2_O_2_]_e_ | Total extra-mitochondrial hydrogen peroxide concentration | M |

**Table S1.2.** Dissociation Constants at 37 ºC, I = 0.17 M

| Parameter | Definition | Value | Reference |
| --- | --- | --- | --- |
| $K_{ATP}^{H}$ | Proton ATP binding constant | 10^-6.53^ M | [*^5^*](#_ENREF_5) |
| $K_{ATP}^{Na}$ | Sodium ATP binding constant | 10^-1.14^ M | [*^5^*](#_ENREF_5) |
| $K_{ATP}^{K}$ | Potassium ATP binding constant | 10^-0.99^ M | [*^5^*](#_ENREF_5) |
| $K_{ATP}^{Mg}$ | Magnesium ATP binding constant | 10^-3.92^ M | [*^5^*](#_ENREF_5) |
| $K_{ATP}^{Ca}$ | Calcium ATP binding constant | 10^-3.89^ M | [*^5^*](#_ENREF_5) |
| $K_{ADP}^{H}$ | Proton ADP binding constant | 10^-6.36^ M | [*^5^*](#_ENREF_5) |
| $K_{ADP}^{Na}$ | Sodium ADP binding constant | 10^-0.98^ M | [*^5^*](#_ENREF_5) |
| $K_{ADP}^{K}$ | Potassium ADP binding constant | 10^-0.87^ M | [*^5^*](#_ENREF_5) |
| $K_{ADP}^{Mg}$ | Magnesium ADP binding constant | 10^-3.02^ M | [*^5^*](#_ENREF_5) |
| $K_{ADP}^{Ca}$ | Calcium ADP binding constant | 10^-2.82^ M | [*^5^*](#_ENREF_5) |
| $K_{Pi}^{H}$ | Proton Pi binding constant | 10^-6.66^ M | [*^5^*](#_ENREF_5) |
| $K_{pi}^{Na}$ | Sodium Pi binding constant | 10^-0.61^ M | [*^5^*](#_ENREF_5) |
| $K_{Pi}^{K}$ | Potassium Pi binding constant | 10^-0.41^ M | [*^6^*](#_ENREF_6) |
| $K_{Pi}^{Mg}$ | Magnesium Pi binding constant | 10^-1.47^ M | [*^7^*](#_ENREF_7) |
| $K_{Pi}^{Ca}$ | Calcium Pi binding constant | 10^-2.27^ M | [*^7^*](#_ENREF_7) |
| $K_{EGTA}^{H}$ | 1st proton EGTA binding constant | 10^-9.56^ M | [*^8^*](#_ENREF_8) |
| $K_{HEGTA}^{H}$ | 2nd proton EGTA binding constant | 10^-8.94^ M | [*^8^*](#_ENREF_8) |
| $K_{EGTA}^{K}$ | Potassium EGTA binding constant | 10^-1.20^ M | [*^8^*](#_ENREF_8) |
| $K_{EGTA}^{Mg}$ | Magnesium EGTA binding constant | 10^-5.17^ M | [*^8^*](#_ENREF_8) |
| $K_{EGTA}^{Ca}$ | Calcium EGTA binding constant | 10^-10.9^ M | [*^8^*](#_ENREF_8) |

**Table S1.3.** Physiochemical and General Model Parameters^a^

| Parameter | Definition | Value | Reference |
| --- | --- | --- | --- |
| *R* | Ideal gas constant | 8.314x10^-3^ kJ/K/mol | physical constant |
| *T* | Temperature | 310.15 K | - |
| *F* | Faraday’s constant | 96.487x10^-3^ kJ/mV/mol | physical constant |
| *C_mito_* | IMM capacitance | 1.45x10^-3^ nmol/mg/mV | [*^9^*](#_ENREF_9) |
| *N_tot_* | Total NAD concentration | 3 mM | [*^10^*](#_ENREF_10) |
| *Q_tot_* | Total UQ concentration | 20 mM | [*^2^*](#_ENREF_2) |
| *C_tot_* | Total Cyt C concentration | 200 µM | [*^2^*](#_ENREF_2) |
| *A_tot_* | Total AdN concentration | 10 mM | [*^10^*](#_ENREF_10) |
| *Vol_x_* | Matrix H_2_O volume fraction | 1 µl/mg | b |
| *Vol_i_* | IMS H_2_O volume fraction | 0.11 µl/mg | b |
| *Vol_e_* | buffer H_2_O volume fraction | 10 ml/g | c |
| [*EGTA*] | Extra-mitochondrial EGTA concentration | 2.5 µM | d |

Abbreviations: IMM, inner mitochondrial membrane; NAD, nicotinamide adenine dinucleotide; UQ, ubiquinone; Cyt C, cytochrome c; AdN, adenine nucleotide.

^a^ All thermodynamic data obtained from Li et al. [*^11^*](#_ENREF_11). Additional data is included in this table.

^b^ Approximate mitochondrial matrix and IMS volumes.

^c^ Based on mitochondrial concentration of 0.1 mg/ml.

^d^ Based on 5 µl per 2 ml carrier over of 1 mM EGTA in 40 mg/ml mitochondrial stock solution.

**S2 - Model Differential-Algebraic Equations**

S2.2A - Model Differential Equations (Bioenergetics)

*Mitochondrial Membrane Potential:*

| $\frac{d\Delta\Psi}{dt}=\left(4J_{C1}^{NADH}+J_{C3}+4J_{C4}-nH_{F_{1}F_{O}}J_{F_{1}F_{O}}-J_{ANT}-J_{Hleak}-2J_{MCU}-J_{NCLX}\right)/C_{mito}$ | (2.1) |
| --- | --- |

*Mitochondrial State Variables:*

| $\frac{d[ADP]_{x}}{dt}=\left(J_{ANT}-J_{F_{1}F_{O}}-nQ_{DH}J_{DH}\right)/Vol_{m}$ | (2.2) |
| --- | --- |
| $\frac{d[Pi]_{x}}{dt}=\left(J_{PIC}-J_{F_{1}F_{O}}-nQ_{DH}J_{DH}\right)/Vol_{m}-m_{CaPi}J_{CaPi}$ | (2.3) |
| $\frac{d[NAD]_{x}}{dt}=\left(J_{C1}^{NADH}-nNADH_{DH}J_{DH}\right)/Vol_{m}$ | (2.4) |
| $\frac{d[UQ]_{x}}{dt}=\left((\frac{1}{2})J_{C3}-J_{C2}-J_{C3}^{NADH}\right)/Vol_{m}$ | (2.5) |
| $\frac{d[\mathrm{O}_{2}^{.-}]_{x}}{dt}=\left(\frac{1}{2}J_{C3}^{SO}+J_{C1}^{SO}\right)/Vol_{m}-2J_{SODx}$ | (2.6) |
| $\frac{d[\mathrm{H}_{2}\mathrm{O}_{2}]_{x}}{dt}=J_{C1}^{H2O2}/Vol_{m}+J_{SODx}-J_{H2O2}-J_{H2O2perm}$ | (2.7) |
| $\frac{d[CaPi]}{dt}=J_{CaPi}$ | (2.8) |

*Inter-Membrane Space (IMS) State Variables*:

| $\frac{d[\mathrm{c}^{3+}]_{i}}{dt}=\left(2J_{C4}-J_{C3}^{c3}\right)/Vol_{i}$ | (2.8) |
| --- | --- |

*Extra-Mitochondrial State Variables:*

| $\frac{d[ATP]_{e}}{dt}=J_{ANT}/Vol_{e}-J_{ATPase}$ | (2.9) |
| --- | --- |
| $\frac{d[ADP]_{e}}{dt}=J_{ATPase}-J_{ANT}/Vol_{e}$ | (2.10) |
| $\frac{d[Pi]_{e}}{dt}=J_{Pi}^{Inject}-J_{PiC}/Vol_{e}+J_{ATPase}$ | (2.11) |
| $\frac{d[\mathrm{O}_{2}^{.-}]_{e}}{dt}=\frac{1}{2}J_{C3}^{SO}/Vol_{e}-2J_{SODe}$ | (2.12) |
| $\frac{d[\mathrm{H}_{2}\mathrm{O}_{2}]_{e}}{dt}=J_{SOD}+J_{H2O2perm}Vol_{m}/Vol_{e}$ | (2.13) |

S2.2B - Mitochondrial Conservation Algebraic Equations

The mitochondrial species for adenine nucleotides (AdNs), nicotinamide adenine dinucleotides (NADH), ubiquinone (UQH_2_), and reduced cytochrome c (c^2+^) are conserved in the model. The conservation is implemented by using an algebraic expression to govern the conservation. This is done using a mass matrix with the integration algorithm and setting the appropriate rows equal to a zero-row vector.

| $[ATP]_{m}=A_{tot}-[ADP]_{m}$ | (2.16) |
| --- | --- |
| $[NADH]_{m}=N_{tot}-[NAD]_{m}$ | (2.17) |
| $[UQH_{2}]_{m}=Q_{tot}-[UQ]_{m}$ | (2.18) |
| $[\mathrm{c}^{2+}]_{i}=C_{tot}-[\mathrm{c}^{3+}]_{i}$ | (2.19) |

S2.2C - Model Differential Equations (Cations)

The cation differential equations for the mitochondrial and extra-mitochondrial compartments are derived using the method outlined in Vinnakota et al.[*^12^*](#_ENREF_12). The general method will be presented with the understanding that compartment specific concentrations and transport rates are to be used where appropriate. In some cases, these compartment specific equations will be given. Due to the large expressions resulting from the derivation, the method used to obtain them is presented versus explicitly showing all the terms that enter the differential equations. The equations used in the model are obtained by solving the linear system of equations given in Equation 2.20.

The generalized system of equations relating the cation differential equations is

|  | (2.20) |
| --- | --- |

where $X_{\partial Y}$is the partial derivative of the concentration of *bound* X with respect to Y (Y can equal X) and $\Phi_{X}$is the flux of X into or out of the compartment.

Assuming higher order cation binding is negligible, the partial derivative expressions are defined below where *N_r_* is the number of reactants, *L_i_* is the *i*^th^ ligand (such as ATP, ADP, and Pi), $K_{i}^{j}$is the dissociation constant for the *i*^th^ ligand and *j*^th^ cation couple and *P_i_* is the binding polynomial for the *i*th ligand as originally defined by Alberty [*^13^*](#_ENREF_13).

| $H_{\partial H}=\frac{\partial[H_{bound}]}{\partial[H^{+}]}=\sum_{i=1}^{Nr} \frac{[L_{i}]\left(1+[Na^{+}]/K_{i}^{Na}+[K^{+}]/K_{i}^{K}+[Mg^{2+}]/K_{i}^{Mg}+[Ca^{2+}]/K_{i}^{Ca}\right)}{K_{i}^{H}P_{i}^{2}}$ | (2.21) |
| --- | --- |
| $H_{\partial Na}=\frac{\partial[H_{bound}]}{\partial[Na^{+}]}=-[H^{+}]\sum_{i=1}^{Nr} \frac{[L_{i}]/K_{i}^{H}}{K_{i}^{Na}P_{i}^{2}}$ | (2.22) |
| $H_{\partial K}=\frac{\partial[H_{bound}]}{\partial[K^{+}]}=-[H^{+}]\sum_{i=1}^{Nr} \frac{[L_{i}]/K_{i}^{H}}{K_{i}^{K}P_{i}^{2}}$ | (2.23) |
| $H_{\partial Mg}=\frac{\partial[H_{bound}]}{\partial[Mg^{2+}]}=-[H^{+}]\sum_{i=1}^{Nr} \frac{[L_{i}]/K_{i}^{H}}{K_{i}^{Mg}P_{i}^{2}}$ | (2.24) |
| $H_{\partial Ca}=\frac{\partial[H_{bound}]}{\partial[Ca^{2+}]}=-[H^{+}]\sum_{i=1}^{Nr} \frac{[L_{i}]/K_{i}^{H}}{K_{i}^{Ca}P_{i}^{2}}$ | (2.25) |
| $Na_{\partial H}=\frac{\partial[Na_{bound}]}{\partial[H^{+}]}=-[Na^{+}]\sum_{i=1}^{Nr} \frac{[L_{i}]/K_{i}^{Na}}{K_{i}^{H}P_{i}^{2}}$ | (2.26) |
| $Na_{\partial Na}=\frac{\partial[Na_{bound}]}{\partial[Na^{+}]}=\sum_{i=1}^{Nr} \frac{[L_{i}]\left(1+[H^{+}]/K_{i}^{H}+[K^{+}]/K_{i}^{K}+[Mg^{2+}]/K_{i}^{Mg}+[Ca^{2+}]/K_{i}^{Ca}\right)}{K_{i}^{Na}P_{i}^{2}}$ | (2.27) |
| $Na_{\partial K}=\frac{\partial[Na_{bound}]}{\partial[K^{+}]}=-[Na^{+}]\sum_{i=1}^{Nr} \frac{[L_{i}]/K_{i}^{Na}}{K_{i}^{K}P_{i}^{2}}$ | (2.28) |
|  | (2.29) |
| $Na_{\partial Ca}=\frac{\partial[Na_{bound}]}{\partial[Ca^{2+}]}=-[Na^{+}]\sum_{i=1}^{Nr} \frac{[L_{i}]/K_{i}^{Na}}{K_{i}^{Ca}P_{i}^{2}}$ | (2.30) |
| $K_{\partial H}=\frac{\partial[K_{bound}]}{\partial[H^{+}]}=-[K^{+}]\sum_{i=1}^{Nr} \frac{[L_{i}]/K_{i}^{K}}{K_{i}^{H}P_{i}^{2}}$ | (2.31) |
| $K_{\partial Na}=\frac{\partial[K_{bound}]}{\partial[Na^{+}]}=-[K^{+}]\sum_{i=1}^{Nr} \frac{[L_{i}]/K_{i}^{K}}{K_{i}^{Na}P_{i}^{2}}$ | (2.32) |
| $K_{\partial K}=\frac{\partial[K_{bound}]}{\partial[K^{+}]}=\sum_{i=1}^{Nr} \frac{[L_{i}]\left(1+[H^{+}]/K_{i}^{H}+[Na^{+}]/K_{i}^{Na}+[Mg^{2+}]/K_{i}^{Mg}+[Ca^{2+}]/K_{i}^{Ca}\right)}{K_{i}^{K}P_{i}^{2}}$ | (2.33) |
| $K_{\partial Mg}=\frac{\partial[K_{bound}]}{\partial[Mg^{2+}]}=-[K^{+}]\sum_{i=1}^{Nr} \frac{[L_{i}]/K_{i}^{K}}{K_{i}^{Mg}P_{i}^{2}}$ | (2.34) |
| $K_{\partial Ca}=\frac{\partial[K_{bound}]}{\partial[Ca^{2+}]}=-[K^{+}]\sum_{i=1}^{Nr} \frac{[L_{i}]/K_{i}^{K}}{K_{i}^{Ca}P_{i}^{2}}$ | (2.35) |
| $Mg_{\partial H}=\frac{\partial[Mg_{bound}]}{\partial[H^{+}]}=-[Mg^{2+}]\sum_{i=1}^{Nr} \frac{[L_{i}]/K_{i}^{Mg}}{K_{i}^{H}P_{i}^{2}}$ | (2.36) |
| $Mg_{\partial Na}=\frac{\partial[Mg_{bound}]}{\partial[Na^{+}]}=-[Mg^{2+}]\sum_{i=1}^{Nr} \frac{[L_{i}]/K_{i}^{Mg}}{K_{i}^{Na}P_{i}^{2}}$ | (2.37) |
| $Mg_{\partial K}=\frac{\partial[Mg_{bound}]}{\partial[K^{+}]}=-[Mg^{2+}]\sum_{i=1}^{Nr} \frac{[L_{i}]/K_{i}^{Mg}}{K_{i}^{K}P_{i}^{2}}$ | (2.38) |
| $Mg_{\partial Mg}=\frac{\partial[Mg_{bound}]}{\partial[Mg^{2+}]}=\sum_{i=1}^{Nr} \frac{[L_{i}]\left(1+[H^{+}]/K_{i}^{H}+[Na^{+}]/K_{i}^{Na}+[K^{+}]/K_{i}^{K}+[Ca^{2+}]/K_{i}^{Ca}\right)}{K_{i}^{Mg}P_{i}^{2}}$ | (2.39) |
| $Mg_{\partial Ca}=\frac{\partial[Mg_{bound}]}{\partial[Ca^{2+}]}=-[Mg^{2+}]\sum_{i=1}^{Nr} \frac{[L_{i}]/K_{i}^{Mg}}{K_{i}^{Ca}P_{i}^{2}}$ | (2.40) |
| $Ca_{\partial H}=\frac{\partial[Ca_{bound}]}{\partial[H^{+}]}=-[Ca^{2+}]\sum_{i=1}^{Nr} \frac{[L_{i}]/K_{i}^{Ca}}{K_{i}^{H}P_{i}^{2}}$ | (2.41) |
| $Ca_{\partial Na}=\frac{\partial[Ca_{bound}]}{\partial[Na^{+}]}=-[Ca^{2+}]\sum_{i=1}^{Nr} \frac{[L_{i}]/K_{i}^{Ca}}{K_{i}^{Na}P_{i}^{2}}$ | (2.42) |
| $Ca_{\partial K}=\frac{\partial[Ca_{bound}]}{\partial[K^{+}]}=-[Ca^{2+}]\sum_{i=1}^{Nr} \frac{[L_{i}]/K_{i}^{Ca}}{K_{i}^{K}P_{i}^{2}}$ | (2.43) |
| $Ca_{\partial Mg}=\frac{\partial[Ca_{bound}]}{\partial[Mg^{2+}]}=-[Ca^{2+}]\sum_{i=1}^{Nr} \frac{[L_{i}]/K_{i}^{Ca}}{K_{i}^{Mg}P_{i}^{2}}$ | (2.44) |
| $Ca_{\partial Ca}=\frac{\partial[Ca_{bound}]}{\partial[Ca^{2+}]}=\sum_{i=1}^{Nr} \frac{[L_{i}]\left(1+[H^{+}]/K_{i}^{H}+[Na^{+}]/K_{i}^{Na}+[K^{+}]/K_{i}^{K}+[Mg^{2+}]/K_{i}^{Mg}\right)}{K_{i}^{Ca}P_{i}^{2}}$ | (2.45) |
| $P_{i}=1+[H^{+}]/K_{i}^{H}+[Na^{+}]/K_{i}^{Na}+[K^{+}]/K_{i}^{K}+[Mg^{2+}]/K_{i}^{Mg}+[Ca^{2+}]/K_{i}^{Ca}$ | (2.46) |

*Additional Buffering in the Mitochondrial Compartment*

To account for additional buffering not attributed to metabolites and substrates in the mitochondrial compartment, equations 2.21, and 2.45 are modified by adding the following terms:

| $H_{\partial H}=H_{\partial H}+\frac{[B_{H}]}{K_{H}\left(1+[H^{+}]/K_{H}\right)^{2}}$ | (2.47) |
| --- | --- |
| $Ca_{\partial Ca}=Ca_{\partial Ca}+\frac{[B_{Ca}]}{K_{Ca}\left(1+[Ca^{2+}]/K_{Ca}\right)^{2}}$ | (2.48) |

**Table S2.1.** Additional Mitochondrial Buffering Parameters

| Parameter | Definition | Value | Reference |
| --- | --- | --- | --- |
| [B_H_] | Total binding sites | 20 mM | [*^1^*](#_ENREF_1) |
| K_H_ | H^+^ binding constant | 100 nM | [*^1^*](#_ENREF_1) |
| [*B_Ca_*] | Total calcium binding sites | 15 mM | a |
| *K_Ca_* | Ca^2+^ binding constant | 1 µM | a |

^a^ Fit to data presented in the main paper.

*Additional Buffering in the Extra-Mitochondrial Compartment*

Both MOPS and EGTA were included in the incubation medium for the conditions simulated in the main paper. Each of these reagents buffers H^+^s, and EGTA also buffers Ca^2+^. Moreover, EGTA is primarily dibasic in the pH range simulated. Chemical impurities and cation binding to biological membranes also modulates extra-mitochondrial buffering of cations. To account for these additional buffers in the extra-mitochondrial compartment, Eqs. 2.47, 2.25, 2.41 and 2.48 are modified by adding the following terms:

| $H_{\partial H}=H_{\partial H}+\frac{[MOPS]}{K_{MOPS}^{H}\left(1+[H^{+}]/K_{MOPS}^{H}\right)^{2}}+\frac{[EGTA]\left(2[H^{+}]+K_{HEGTA}^{H}\right)\left([Ca^{2+}]+K_{EGTA}^{Ca}\right)}{K_{EGTA}^{Ca}K_{EGTA}^{H}K_{HEGTA}^{H}P_{EGTA}^{2}}$ | (2.49) |
| --- | --- |
| $H_{\partial Ca}=H_{\partial Ca}-\frac{[EGTA][H^{+}]\left([H^{+}]+K_{HEGTA}^{H}\right)}{K_{EGTA}^{Ca}K_{EGTA}^{H}K_{HEGTA}^{H}P_{EGTA}^{2}}$ | (2.50) |
| $Ca_{\partial H}=Ca_{\partial H}-\frac{[EGTA][Ca^{2+}]\left(2[H^{+}]+K_{HEGTA}^{H}\right)}{K_{EGTA}^{Ca}K_{EGTA}^{H}K_{HEGTA}^{H}P_{EGTA}^{2}}$ | (2.51) |
| $Ca_{\partial Ca}=Ca_{\partial Ca}+\frac{[EGTA]\left([H^{+}]^{2}+[H^{+}]K_{HEGTA}^{H}+K_{EGTA}^{H}K_{HEGTA}^{H}\right)}{K_{EGTA}^{Ca}K_{EGTA}^{H}K_{HEGTA}^{H}P_{EGTA}^{2}}+\frac{[B_{Ca}]_{e}}{K_{Cae}\left(1+[Ca^{2+}]/K_{Cae}\right)^{2}}$ | (2.52) |
| $P_{EGTA}=1+[H^{+}]/K_{EGTA}^{H}+[H^{+}]^{2}/K_{HEGTA}^{H}/K_{EGTA}^{H}+[Ca^{2+}]/K_{EGTA}^{Ca}$ | (2.53) |

**Table S2.2.** Additional Extra-Mitochondrial Buffering Parameters

| Parameter | Definition | Value | Reference |
| --- | --- | --- | --- |
| [*B_Ca_*]*_e_* | Additional Buffer Ca^2+^ Binding Sites | 400 µM | a |
| *K_Cae_* | Ca^2+^ binding constant | 150 µM | a |

^a^ Fit to data shown in Figure S2.1.

**Figure S2.1. Extra-mitochondrial calcium buffering power is small but significant.** The extra-mitochondrial calcium buffering power of respiration buffer in the presence of 0.1 mg/ml freeze thawed mitochondria was quantified by determining the change in free calcium divided by the total calcium added. This was done by adding 2 µM CaCl_2_ while monitoring the free calcium using 1 µM calcium green.

*Flux Terms*

The terms representing flux into a given compartment are defined as

| $\Phi_{H}=-\sum_{i=1}^{Nr} \frac{\partial[H_{bound}]}{\partial[L_{i}]}\frac{d[L_{i}]}{dt}+\sum_{k=1}^{Nk} n_{k}J_{k}+J_{t}^{H}$ | (2.54) |
| --- | --- |
| $\Phi_{Na}=-\sum_{i=1}^{Nr} \frac{\partial[Na_{bound}]}{\partial[L_{i}]}\frac{d[L_{i}]}{dt}+J_{t}^{Na}$ | (2.55) |
| $\Phi_{K}=-\sum_{i=1}^{Nr} \frac{\partial[K_{bound}]}{\partial[L_{i}]}\frac{d[L_{i}]}{dt}+J_{t}^{K}$ | (2.56) |
| $\Phi_{Mg}=-\sum_{i=1}^{Nr} \frac{\partial[Mg_{bound}]}{\partial[L_{i}]}\frac{d[L_{i}]}{dt}+J_{t}^{Mg}$ | (2.57) |
| $\Phi_{Ca}=-\sum_{i=1}^{Nr} \frac{\partial[Ca_{bound}]}{\partial[L_{i}]}\frac{d[L_{i}]}{dt}+J_{t}^{Ca}$ | (2.58) |

where *N_k_* is the number of reactions, *n_k_* is the stoichiometric coefficient of *k*^th^ reaction, *J_k_* is the *k*^th^ reaction rate and $J_{t}^{i}$ is the *i*^th^ cation transport rate into the compartment.

The ligand dependent partial derivative expressions are defined as

| $\frac{\partial[H_{bound}]}{\partial[L_{i}]}=[H^{+}]\sum_{i=1}^{Nr} \frac{1}{K_{i}^{H}P_{i}}$ | (2.59) |
| --- | --- |
| $\frac{\partial[Na_{bound}]}{\partial[L_{i}]}=[Na^{+}]\sum_{i=1}^{Nr} \frac{1}{K_{i}^{Na}P_{i}}$ | (2.60) |
| $\frac{\partial[K_{bound}]}{\partial[L_{i}]}=[K^{+}]\sum_{i=1}^{Nr} \frac{1}{K_{i}^{K}P_{i}}$ | (2.61) |
| $\frac{\partial[Mg_{bound}]}{\partial[L_{i}]}=[Mg^{2+}]\sum_{i=1}^{Nr} \frac{1}{K_{i}^{Mg}P_{i}}$ | (2.62) |
| $\frac{\partial[Ca_{bound}]}{\partial[L_{i}]}=[Ca^{2+}]\sum_{i=1}^{Nr} \frac{1}{K_{i}^{Ca}P_{i}}$ | (2.63) |

*Compartment Reactions and Fluxes*

The generation of protons by biochemical reactions in the mitochondria is defined as

| $\sum_{k=1}^{Nk} n_{k}J_{k}=J_{DH}$ | (2.64) |
| --- | --- |

This term is zero in the extra-mitochondrial compartment.

The transport of cations into the mitochondrial compartment are defined as

| $\begin{matrix} J_{t}^{H}=+(3J_{DH}-5J_{C1}^{NADH}-J_{C3}-4J_{4}-(nH_{F1FO}-1)J_{F1FO}+2J_{Pic}+J_{Hleak}+J_{KHE}+J_{NHE})/vol_{m} \\ +2(n_{Capi}-m_{Capi})J_{CaPi} \end{matrix}$ | (2.41) |
| --- | --- |
| $J_{t}^{Na}=(3J_{NCLX}-J_{NHE})/vol_{m}$ | (2.42) |
| $J_{t}^{K}=J_{KHE}/vol_{m}$ | (2.43) |
| $J_{t}^{Mg}=0$ | (2.44) |
| $J_{t}^{Ca}=(J_{MCU}-J_{NCLX})/vol_{m}-n_{CaPi}J_{CaPi}$ | (2.45) |

The transport terms for the extra-mitochondrial compartment are the negative of their mitochondrial counterparts.

**S3: Model Rate Equations**

Each reaction rate and transport mechanism in the model is presented below. First, the electron transport related reaction rates are shown. Next, the oxidative phosphorylation related reaction rates and transport mechanisms are discussed. Finally, the cation transport and ROS scavenging mechanisms are presented.

S3A - Electron Transport Related Reaction Rates

Substrate oxidation and the electron transport system are based on the rate equations presented in Beard [*^10^*](#_ENREF_10). The mitochondrial dehydrogenase rate (substrate oxidation rate) will be introduced first, followed by the electron transport system rate equations and finally the passive proton leak rate equation is presented.

*Mitochondrial Dehydrogenases*

The biochemical equation for the mitochondrial dehydrogenase is defined as shown below. The equations assume that calcium activates TCA and bring 1 ubiquinol for every 4 NADH (complete span of TCA cycle) with no complex II activity (complex II is proportional to mitochondrial dehydrogenases) for sodium pyruvate substrate.

$NAD_{x}^{+}\rightleftarrows NADH_{x}+H_{x}^{+}$.

The rate expression used in the model is

$J_{\text{DH}}=X_{\text{DH}}\frac{\left(\frac{[ADP]_{x}[Pi]_{x}}{[ATP]_{x}}\right)^{n_{DH}}}{\left(\frac{[ADP]_{x}[Pi]_{x}}{[ATP]_{x}}\right)^{n_{DH}}+KA_{DH}^{n_{DH}}}\frac{[NAD]_{x}}{[NAD]_{x}+KN_{DH}[NADH]_{x}}$.

**Table S3.1.** Mitochondrial Dehydrogenase Parameters^b^

| Parameter | Definition | Value | Reference |
| --- | --- | --- | --- |
| *X_DH_* | Dehydrogenase activity | 248 mol/lmito/s | a |
| *KN_DH_* | NADH/NAD^+^ feedback constant | 0.1432 | a |
| *KA_DH_* | ATPase feedback constant | 0.0591 M | a |
| *n_DH_* | ATPase feedback Hill coefficient | 1.151 | a |

^a^ Adjustable parameter fit from data in Vinnakota et al.[*^14^*](#_ENREF_14).

*NADH-ubiquinone oxidoreductase: Complex I*

The biochemical equation for Complex I is defined as

$UQ_{x}+NADH_{x}+5H_{x}^{+}\rightleftarrows NAD_{x}^{+}+UQH_{2x}+4H_{e}^{+}$.

For model details, see [*^15^*](#_ENREF_15). In brief, the model is a five-state model that includes the minimal components necessary to simulate NADH-quinone oxidoreductase activity as a function of pH and mitochondrial membrane potential (ΔΨ) and also the detailed redox biochemistry required to simulate ROS generation by both the FMN and the SQ sites. The model consists of numerous binding polynomials that represent various combinations free, substrate-bound, product-bound, and protonated states and characterizes the multiple redox states of the enzyme. In addition, the thermodynamic equations are corrected for pH, temperature, and ionic strength. The model equations consist of about a hundred supporting equations that culminate into the following matrix equation describing the steady state fractional occupancies of the differing redox states:

$$\left[\begin{matrix} -\left(k_{0\to1}+k_{0\to2}\right) & k_{1\to0} & k_{2\to0} & 0 & 0 \\ k_{0\to1} & -(k_{1\to0}+k_{1\to2}+k_{1\to3}) & k_{2\to1} & k_{3\to1} & 0 \\ k_{0\to2} & k_{1\to2} & -(k_{2\to0}+k_{2\to1}+k_{2\to3}+k_{2\to4}) & k_{3\to2} & k_{4\to2} \\ 0 & k_{1\to3} & k_{2\to3} & -(k_{3\to1}+k_{3\to2}+k_{3\to4}) & k_{4\to3} \\ 0 & 0 & k_{2\to4} & k_{3\to4} & -\left(k_{4\to2}+k_{4\to3}\right) \\ 1 & 1 & 1 & 1 & 1 \end{matrix}\right]\left[\begin{matrix} S0 \\ S1 \\ S2 \\ S3 \\ S4 \end{matrix}\right]=\left[\begin{matrix} 0 \\ 0 \\ 0 \\ 0 \\ 0 \\ 1 \end{matrix}\right]$$

For definitions of the individual rate constants and their constituents (*kN*’s, *kSO*’s, and *kH*’s), see the original article. After solving for the state occupancy vector, the rate of NADH oxidation is given as:

$J_{C1}^{NADH}=E_{totC1}\left(kN_{f}^{0\to2}S0+kN_{f}^{1\to3}S1+\left(kN_{f}^{2\to4}-kN_{r}^{0\to2}\right)S2-kN_{r}^{1\to3}S3-kN_{r}^{2\to4}S4\right)$.

The rate of superoxide generation is given as:

$J_{C1}^{SO}=E_{totC1}\left(-kSO_{r}^{1\to0}S0+\left(kSO_{f}^{1\to0}-kSO_{r}^{2\to1}\right)S1+\left(kSO_{f}^{2\to1}-kSO_{r}^{3\to2}\right)S2+\left(kSO_{f}^{3\to2}-kSO_{r}^{4\to3}\right)S3+kSO_{f}^{4\to3}S4\right)$.

The rate of hydrogen peroxide generation is given as:

$J_{C1}^{H_{2}O_{2}}=E_{totC1}\left(-kH_{r}^{2\to0}S0-kH_{r}^{3\to1}S1+\left(kH_{f}^{2\to0}-kH_{r}^{4\to2}\right)S2+kH_{f}^{3\to1}S3+kH_{f}^{4\to2}S4\right)$.

And for numerical stability, the rate of UQ reduction is coupled to the above rates such that:

$J_{C1}^{UQ}=J_{C1}^{NADH}-J_{C1}^{SO}/2-J_{C1}^{H_{2}O_{2}}$.

Below is an updated list of parameters.

**Table S3.2.** NADH-Ubiquinone Oxidoreductase Parameters^a^

| Parameter | Definition | Value | Reference |
| --- | --- | --- | --- |
| *Structural Parameters* | | | |
| $E_{m}^{0}(FMN/\mathrm{FMNH}_{2})$ | FMN/FMNH_2_ midpoint potential | 55.14 mV | [*^16^*](#_ENREF_16)^b^ |
| $E_{m}^{0}(FMN/\mathrm{FMNH}^{.-})$ | FMN/FMNH^.-^ midpoint potential | 23.54 mV | [*^16^*](#_ENREF_16)^b^ |
| $E_{m}^{0}(FMNH^{.-}/\mathrm{FMNH}_{2})$ | FMNH^.-^/FMNH_2_ midpoint potential | 86.74 mV | [*^16^*](#_ENREF_16)^b^ |
| $pK_{FMNH_{2}}$ | FMNH_2_ pK | 7.1 | [*^16^*](#_ENREF_16)^b^ |
| $pK_{FMNH^{.-}}$ | FMNH^.-^ pK | 7.9 | [*^16^*](#_ENREF_16)^b^ |
| $E_{m}^{0}(\mathrm{N}2_{\mathrm{ox}}/\mathrm{N}2_{\mathrm{red}})$ | N2_ox_/N2_red_ midpoint potential | -90 mV | [*^17^*](#_ENREF_17)^c^ |
| $pK_{N2^{ox}}$ | N2_ox_ pK | 6 | [*^17^*](#_ENREF_17)^c^ |
| $pK_{N2^{red}}$ | N2_red_ pK | 8.5 | [*^17^*](#_ENREF_17)^c^ |
| *Kinetic Parameters* | | | |
| *E_totC1_* | Total Complex I content | 417 pmol/mg | d |
| $K_{NADH}^{ox}$ | NADH dissociation constant for oxidized FMN | 4.61E-05 M | [*^15^*](#_ENREF_15) |
| $K_{NAD^{+}}^{ox}$ | NAD^+^ dissociation constant for oxidized FMN | 7.05E-04 M | [*^15^*](#_ENREF_15) |
| $K_{NADH}^{red}$ | NADH dissociation constant for reduced FMN | 4.99E-04 M | [*^15^*](#_ENREF_15) |
| $K_{NAD^{+}}^{red}$ | NAD^+^ dissociation constant for reduced FMN | 1.18E-05 M | [*^15^*](#_ENREF_15) |
| $K_{NADH}^{rad}$ | NADH dissociation constant for FMN radical | 1.00E+00 M | [*^15^*](#_ENREF_15) |
| $K_{NAD^{+}}^{rad}$ | NAD^+^ dissociation constant for FMN radical | 1.54E-04 M | [*^15^*](#_ENREF_15) |
| $K_{Q_{10}H_{2}}$ | Q_10_H_2_ dissociation constant | 1.00E-01 M | [*^15^*](#_ENREF_15) |
| $K_{Q_{10}}$ | Q_10_ dissociation constant | 1.75E-02 M | [*^15^*](#_ENREF_15) |
| $K_{Q_{10}}^{stability}$ | Q_10_ stability constant | 1.00E+01 | [*^15^*](#_ENREF_15) |
| $k_{f,0\to2}^{NADH}$ | NADH oxidation rate for state 0 | 1.18E+05 s^-1^ | [*^15^*](#_ENREF_15) |
| $k_{f,2\to4}^{NADH}$ | NADH oxidation rate for state 2 | 1.12E+04 s^-1^ | [*^15^*](#_ENREF_15) |
| $k_{f,1\to3}^{NADH}$ | NADH oxidation rate for state 1 | 2.77E+02 s^-1^ | [*^15^*](#_ENREF_15) |
| $k_{f,2\to0}^{Q}$ | Q reduction rate for state 2 | 3.49E+05 s^-1^ | [*^15^*](#_ENREF_15) |
| $k_{f,4\to2}^{Q}$ | Q reduction rate for state 4 | 4.67E+12 s^-1^ | [*^15^*](#_ENREF_15) |
| $k_{f,3\to1}^{Q}$ | Q reduction rate for state 3 | 5.20E+02 s^-1^ | [*^15^*](#_ENREF_15) |
| *β* | Charge translocation parameter | 5.00E-01 | [*^15^*](#_ENREF_15) |
| $pK_{Nsite}$ | NADH oxidase site pK | 7.39 | [*^15^*](#_ENREF_15) |
| $pK_{Qsite}$ | Q reductase site pK | 6.41 | [*^15^*](#_ENREF_15) |
| *ROS Parameters* | | | |
| $k_{f,1\to0}^{SQ^{.-}/O_{2}^{.-}}$ | Superoxide production rate from semiquinone for state 1 | 1.67E+09 M^-1^s^-1^ | [*^15^*](#_ENREF_15) |
| $k_{fa,2\to1}^{SQ^{.-}/O_{2}^{.-}}$ | Superoxide production rate from semiquinone for state 2a | 2.06E-03 M^-1^s^-1^ | [*^15^*](#_ENREF_15) |
| $k_{fb,2\to1}^{SQ^{.-}/O_{2}^{.-}}$ | Superoxide production rate from semiquinone for state 2b | 2.07E-03 M^-1^s^-1^ | [*^15^*](#_ENREF_15) |
| $k_{fa,3\to2}^{SQ^{.-}/O_{2}^{.-}}$ | Superoxide production rate from semiquinone for state 3a | 1.14E-05 M^-1^s^-1^ | [*^15^*](#_ENREF_15) |
| $k_{fb,3\to2}^{SQ^{.-}/O_{2}^{.-}}$ | Superoxide production rate from semiquinone for state 3b | 2.40E-09 M^-1^s^-1^ | [*^15^*](#_ENREF_15) |
| $k_{f,4\to3}^{SQ^{.-}/O_{2}^{.-}}$ | Superoxide production rate from semiquinone for state 4 | 2.16E-07 M^-1^s^-1^ | [*^15^*](#_ENREF_15) |
| $k_{f,2\to1}^{FMNH_{2}/O_{2}^{.-}}$ | Superoxide production rate from FMNH_2_ for state 2 | 4.52E+05 M^-1^s^-1^ | [*^15^*](#_ENREF_15) |
| $k_{fa,3\to2}^{FMNH_{2}/O_{2}^{.-}}$ | Superoxide production rate from FMNH_2_ for state 3a | 5.38E-04 M^-1^s^-1^ | [*^15^*](#_ENREF_15) |
| $k_{fb,3\to2}^{FMNH_{2}/O_{2}^{.-}}$ | Superoxide production rate from FMNH_2_ for state 3b | 2.83E-05 M^-1^s^-1^ | [*^15^*](#_ENREF_15) |
| $k_{f,4\to3}^{FMNH_{2}/O_{2}^{.-}}$ | Superoxide production rate from FMNH_2_ for state 4 | 1.40E+05 M^-1^s^-1^ | [*^15^*](#_ENREF_15) |
| $k_{f,1\to0}^{FMNH^{.-}/O_{2}^{.-}}$ | Superoxide production rate from FMNH^.-^ for state 1 | 6.81E+07 M^-1^s^-1^ | [*^15^*](#_ENREF_15) |
| $k_{fa,2\to1}^{FMNH^{.-}/O_{2}^{.-}}$ | Superoxide production rate from FMNH^.-^ for state 2a | 4.04E-11 M^-1^s^-1^ | [*^15^*](#_ENREF_15) |
| $k_{fb,2\to1}^{FMNH^{.-}/O_{2}^{.-}}$ | Superoxide production rate from FMNH^.-^ for state 2b | 1.19E-07 M^-1^s^-1^ | [*^15^*](#_ENREF_15) |
| $k_{f,3\to2}^{FMNH^{.-}/O_{2}^{.-}}$ | Superoxide production rate from FMNH^.-^ for state 3 | 1.40E-02 M^-1^s^-1^ | [*^15^*](#_ENREF_15) |
| $k_{f,2\to0}^{FMNH_{2}/\mathrm{H}_{2}O_{2}}$ | Hydrogen peroxide production rate from FMNH_2_ for state 2 | 7.82E-07 M^-1^s^-1^ | [*^15^*](#_ENREF_15) |
| $k_{fa,3\to1}^{FMNH_{2}/\mathrm{H}_{2}O_{2}}$ | Hydrogen peroxide production rate from FMNH_2_ for state 3a | 2.12E+06 M^-1^s^-1^ | [*^15^*](#_ENREF_15) |
| $k_{fb,3\to1}^{FMNH_{2}/\mathrm{H}_{2}O_{2}}$ | Hydrogen peroxide production rate from FMNH_2_ for state 3b | 1.76E+06 M^-1^s^-1^ | [*^15^*](#_ENREF_15) |
| $k_{f,4\to2}^{FMNH_{2}/\mathrm{H}_{2}O_{2}}$ | Hydrogen peroxide production rate from FMNH_2_ for state 4 | 6.21E+01 M^-1^s^-1^ | [*^15^*](#_ENREF_15) |

^a^ Updated from Bazil et al.[*^15^*](#_ENREF_15). ^b^ Refit from data in Figures 1, 2 and 3 of Sled et al. [*^16^*](#_ENREF_16). ^c^ Refit from data in Figure 1 of Ingledew and Ohnishi [*^17^*](#_ENREF_17). ^d^ Adjustable parameter fit from data in Vinnakota et al.[*^14^*](#_ENREF_14).

*Succinate-ubiquinone oxidoreductase: Complex II*

The biochemical equation for Complex II is defined as

$UQ_{x}+SUC_{x}\rightleftarrows FUM_{x}+UQH_{2x}$.

The rate expression used in the model for sodium pyruvate simulation which is proportional to mitochondrial dehydrogenases flux (J_DH_). It assumes 1 ubiquniol produced from TCA cycle:

$J_{C2}=J_{DH}$.

The rate expression used in the model for succinate plus rotenone is

$J_{C2}=X_{C2}(1-[UQH_{2}]_{x}/[UQ]_{x}/K_{C3}^{eq})$.

**Table S3.2.** Complex II Parameters

| Parameter | Definition | Value | Reference |
| --- | --- | --- | --- |
| *X_C2_* | Succinate dehydrogenase activity | 850 nmol/mg/min | a |
| $K_{C2}^{eq}$ | Equilibrium constant | 7500 | a |

^a^ Fit to leak and ADP-stimulated rates for the 1 mM EGTA conditions.

*Ubiquinol-cytochrome-c oxidoreductase: Complex III*

The biochemical equation for Complex III is defined as

$UQH_{2x}+2c_{i}^{3+}+2H_{x}^{+}\rightleftarrows UQ_{x}+2c_{i}^{2+}+4H_{e}^{+}$.

For model details, see Bazil et al.[*^2^*](#_ENREF_2). In brief, the model is a six-state model that simulates quinol-cytochrome c oxidoreductase activity as a function of pH and ΔΨ, as well as, the redox biochemistry involved in superoxide formation. Electrons are added or removed from the complex either one at a time at the Qp-site or two at a time at the Qn-site. The last row in the matrix equation given below is a conservation equation to fix the sum of the state occupancies to equal one.

The model consists of numerous binding polynomials that represent various combinations free, substrate-bound, product-bound, and protonated states and characterizes the multiple redox states of the enzyme. In addition, the thermodynamic equations are corrected for pH, temperature, and ionic strength. The model equations consist of dozens of supporting equations that culminate into the following matrix equation describing the steady state fractional occupancies of the differing redox states:

$$\left[\begin{matrix} -(\mathrm{k}_{01}+\mathrm{k}_{02}) & k_{10} & k_{20} & 0 & 0 & 0 \\ k_{01} & -(\mathrm{k}_{10}+\mathrm{k}_{12}+\mathrm{k}_{13}) & k_{21} & k_{31} & 0 & 0 \\ k_{02} & k_{12} & -(\mathrm{k}_{20}+\mathrm{k}_{21}+\mathrm{k}_{23}) & k_{32} & k_{42} & 0 \\ 0 & k_{13} & k_{23} & -(\mathrm{k}_{31}+\mathrm{k}_{32}+k_{34}) & k_{43} & k_{53} \\ 0 & 0 & k_{24} & k_{34} & -(\mathrm{k}_{42}+k_{43}+k_{45}) & k_{54} \\ 0 & 0 & 0 & k_{35} & k_{45} & -(k_{53}+k_{54}) \\ 1 & 1 & 1 & 1 & 1 & 1 \end{matrix}\right]\left[\begin{matrix} E_{0} \\ E_{1} \\ E_{2} \\ E_{3} \\ E_{4} \\ E_{5} \end{matrix}\right]=\left[\begin{matrix} 0 \\ 0 \\ 0 \\ 0 \\ 0 \\ 1 \end{matrix}\right]$$

For definitions of the individual rate constants, their constituents (e.g., *k_QH2c3+_*’s, *k_QH2_*’s, and *k_SO_*’s), and other model terms, see the original article. After solving for the state occupancy vector, the rate of cytochrome c reduction is given as:

$J_{c}=B_{f}\sum_{i} k_{i,i+1}E_{i}\sum_{\forall r\notin\left\{SQ_{p}\right\}} s_{r}^{i}-B_{r}\sum_{i} k_{i+1,i}E_{i+1}\sum_{\forall r\in\left\{SQ_{p}\right\}} s_{r}^{i+1}$.

The rate of superoxide generation is given as:

$J_{SO}=[O_{2}]kf_{SO}\sum_{i} E_{i}\sum_{\forall r\in\left\{SQ_{p}\right\}} s_{r}^{i}-B_{Q}[O_{2}^{.-}]kr_{SO}\sum_{i} E_{i+1}\sum_{\forall r\notin\left\{SQ_{p}\right\}} s_{r}^{i+1}$.

And for numerical stability, the rate of ubiquinol oxidation is coupled to the above rates such that:

$J_{Qp}=J_{c}$.

**Table S3.3.** Ubiquinol-Cytochrome-c Oxidoreductase Parameters^a^

| Parameter | Definition | Value^a^ | Units | Reference |
| --- | --- | --- | --- | --- |
| *E_totC3_* | Total Complex III content | 417 | pmol/mg | d |
| *K_A_ values* | | | | |
| $K_{A}^{ISPox}$ | Oxidized Rieske iron-sulfur cluster acidic group protonation constant | 10^-6.6^ | - | [*^18^*](#_ENREF_18) |
| $K_{B}^{ISPox}$ | Oxidized Rieske iron-sulfur cluster basic group protonation constant | 10^-9.2^ | - | [*^18^*](#_ENREF_18) |
| $K_{A}^{b_{L}ox}$ | Oxidized heme b_L_ protonation constant | 10^-5.9^ | - | [*^19^*](#_ENREF_19) |
| $K_{A}^{b_{L}red}$ | Reduced heme b_L_ protonation constant | 10^-7.9^ | - | [*^19^*](#_ENREF_19) |
| $K_{A}^{b_{H}ox}$ | Oxidized heme b_H_ protonation constant | 10^-5.7^ | - | [*^19^*](#_ENREF_19) |
| $K_{A}^{b_{H}red}$ | Reduced heme b_H_ protonation constant | 10^-7.7^ | - | [*^19^*](#_ENREF_19) |
| *Thermodynamic values* | | | | |
| $E_{m}^{7}(ISP_{ox}/ISP_{red})$ | Rieske ISP midpoint potential | 300 | mV | [*^20^*](#_ENREF_20) |
| $E_{m}^{0}(b_{L}^{3+}/b_{L}^{2+})$ | Cytochrome b_L_ midpoint potential | -30 (39) | mV | [*^19^*](#_ENREF_19) |
| $E_{m}^{0}(b_{H}^{3+}/b_{H}^{2+})$ | Cytochrome b_H_ midpoint potential | 90 (160) | mV | [*^19^*](#_ENREF_19) |
| $E_{m}^{0}(Q/SQ)_{n}$ | Qn-site semiquinone midpoint potential | -37 | mV | [*^21^*](#_ENREF_21) |
| $E_{m}^{0}(SQ/QH_{2})_{n}$ | Qn-site semiquinone midpoint potential | 92 (954) | mV | [*^21^*](#_ENREF_21) |
| $\beta$ | Fraction of total charge translocation sensed between bL and bH | 0.5 | - | [*^19^*](#_ENREF_19)*,* [*^22-24^*](#_ENREF_22) |
| $\Delta G_{b_{H}/b_{L}}^{Coulomb}$ | b_H_/b_L_ monomeric Coulombic interaction energy | 11.6 | kJ/mol | [*^2^*](#_ENREF_2) |
| $\Delta G_{b_{L}/b_{L}}^{Coulomb}$ | b_L_/b_L_ dimeric Coulombic interaction energy | 5.3 | kJ/mol | [*^2^*](#_ENREF_2) |
| $K_{S}^{Q10}$ | Qp-site stability constant for Q10 | 2.28E-15 | - | [*^2^*](#_ENREF_2) |
| *Q_10_ related constants* | | | | |
|  | Q_10_H_2_ binding constant at Qp-site | 1.6 | mM | [*^25^*](#_ENREF_25) |
|  | Q_10_ binding constant at Qp-site | 1.6 | mM | [*^25^*](#_ENREF_25) |
|  | Q_10_ binding constant at Qn-site | 0.25 | mM | [*^26^*](#_ENREF_26)^b^ |
|  | Q_10_H_2_ binding constant at Qn-site | 2.5 | µM | [*^26^*](#_ENREF_26)^b^ |
| Q_10,tot_ | Q_10_ pool size | 20 | mM | [*^27^*](#_ENREF_27)*,* [*^28^*](#_ENREF_28)^c^ |
| *Superoxide related constants* | | | | |
| $kf_{SO}$ | Superoxide production 2^nd^ order rate constant | 10^10^ | M^-1^s^-1^ | [*^29^*](#_ENREF_29) |
| *Kinetic rate constants* | | | | |
| $kf_{Q_{p}}^{0,1}$ | Quinol oxidation rate for *E_0_* | 1.43E+5 | min^-1^ | [*^2^*](#_ENREF_2) |
| $kf_{Q_{p}}^{1,2}$ | Quinol oxidation rate for *E_1_* | 7.53E+4 | min^-1^ | [*^2^*](#_ENREF_2) |
| $kf_{Q_{p}}^{2,3}$ | Quinol oxidation rate for *E_2_* | 1.90E+5 | min^-1^ | [*^2^*](#_ENREF_2) |
| $kf_{Q_{p}}^{3,4}$ | Quinol oxidation rate for *E_3_* | 1.36E+4 | min^-1^ | [*^2^*](#_ENREF_2) |
| $kf_{Q_{p}}^{4,5}$ | Quinol oxidation rate for *E_4_* | 248 | min^-1^ | [*^2^*](#_ENREF_2) |
| $kf_{Q_{n}}^{2,0}$ | Quinone reduction rate for *E_2_* | 5.52E+11 | min^-1^ | [*^2^*](#_ENREF_2) |
| $kf_{Q_{n}}^{3,1}$ | Quinone reduction rate for *E_3_* | 5.52E+11 | min^-1^ | [*^2^*](#_ENREF_2) |
| $kf_{Q_{n}}^{4,2}$ | Quinone reduction rate for *E_4_* | 4.19E+5 | min^-1^ | [*^2^*](#_ENREF_2) |
| $kf_{Q_{n}}^{5,3}$ | Quinone reduction rate for *E_5_* | 6.78E+5 | min^-1^ | [*^2^*](#_ENREF_2) |
| *Cytochrome c binding constants* | | | | |
| $K_{c^{3+}}$ | c^3+^ binding constant | 1.11E-06 | M | [*^2^*](#_ENREF_2) |
| $K_{c^{2+}}$ | c^2+^ binding constant | 2.49E-06 | M | [*^2^*](#_ENREF_2) |
| ^a^Values are at pH 7 and at 25 °C. Values in parenthesis are at pH 0 and 25 °C. ^b^Values chosen to match reported kinetic and thermodynamic behavior of enzyme. ^c^Estimated by assuming a total mitochondrial Q pool of 5.8 nmol/mg and an inner membrane volume of 290 nl/mg. ^d^ Adjustable parameter fit from data in Vinnakota et al.[*^14^*](#_ENREF_14). | | | | |

*Cytochrome-c oxidase: Complex IV*

The biochemical equation for Complex IV is defined as

$2CytC_{i}^{2+}+\frac{1}{2}O_{2x}+4H_{x}^{+}\rightleftarrows2CytC_{i}^{3+}+H_{2}O+2H_{e}^{+}$.

The equilibrium constant is defined as

$K_{eq}=e^{-\left(\Delta_{r}G_{C4}^{0}-4F\Delta\Psi\right)/RT}\left(\left[H^{+}\right]_{x}^{4}/\left[H^{+}\right]_{e}^{2}\right)$.

The rate expression used in the model is

$J_{C4}=X_{C4}\left(\frac{1}{1+K_{O_{2}}/[O_{2}]_{x}}\right)\left(\frac{[c^{2+}]_{i}^{n}}{[\mathrm{c}^{2+}]_{i}^{n}+K_{M}^{n}\left(1+\beta e^{2\Delta\Psi F/RT}\right)}\right)\left(1-\frac{[\mathrm{c}^{3+}]_{i}^{2}}{[\mathrm{c}^{2+}]_{i}^{2}[O_{2}]_{x}^{1/2}K_{eq}}\right)$.

**Table S3.4.** Cytochrome-c Oxidase Parameters

| Parameter | Definition | Value | Reference |
| --- | --- | --- | --- |
| *X_C4_* | Complex IV activity | 1.2x10^4^ nmol/mg/min | a |
| $\Delta_{r}G_{C4}^{0}$ | Gibb’s free energy of reaction | -202.16 kJ/mol | [*^30^*](#_ENREF_30) |
| $K_{O_{2}}$ | O_2_ binding constant | 1x10^-6^ M | [*^10^*](#_ENREF_10) |
| *K_M_* | c^2+^ binding constant | 162 µM | [*^31^*](#_ENREF_31) |
| *n* | Hill coefficient for c^2+^ | 2 | [*^31^*](#_ENREF_31) |
| *β* | ΔΨ constant | 6.6x10^-6^ | [*^31^*](#_ENREF_31) |

^a^ Adjustable parameter fit from data in Vinnakota et al.[*^14^*](#_ENREF_14).


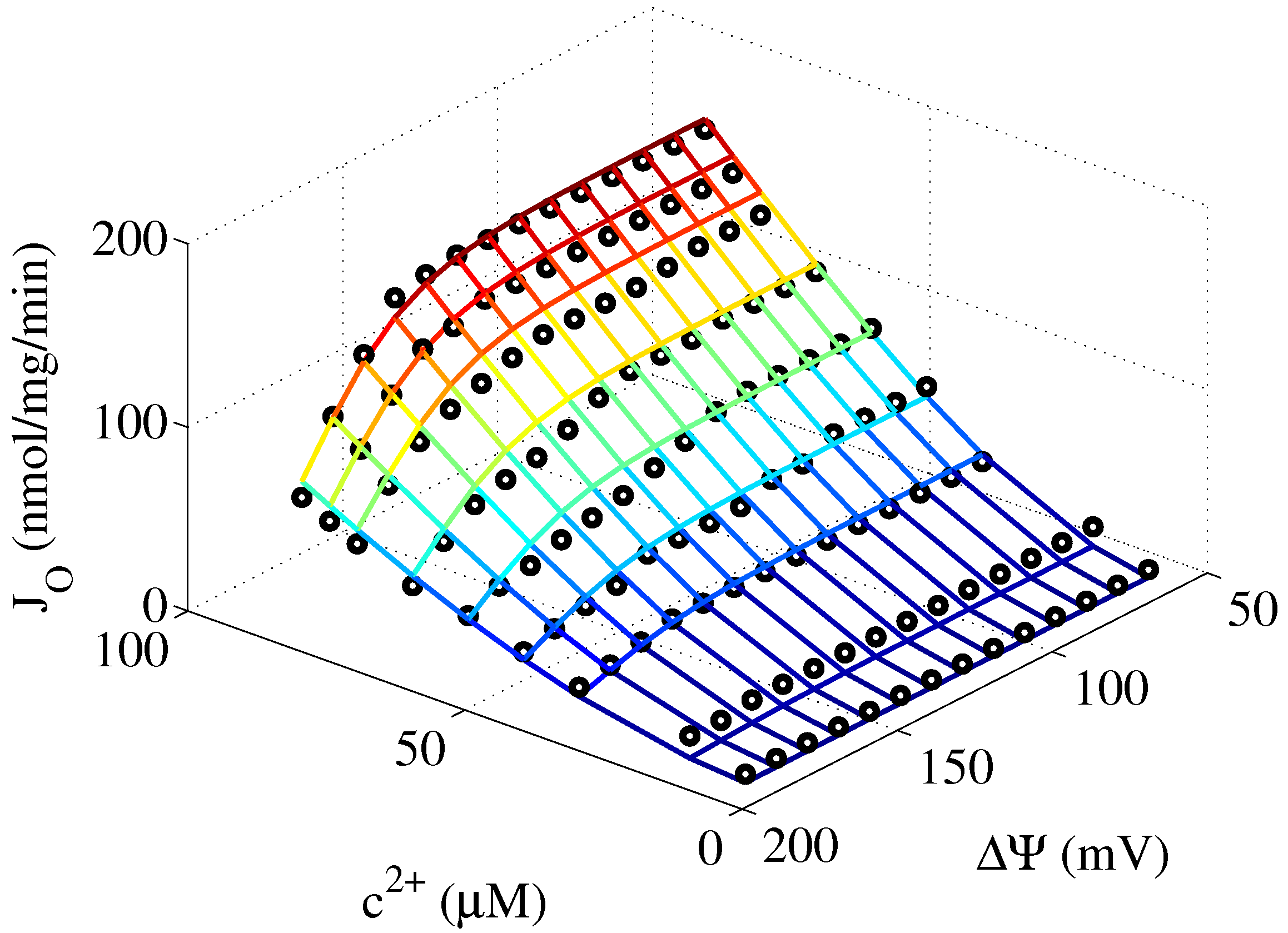


**Figure S3.1. Flux through Complex IV as a function of c^2+^ and ΔΨ.** The data from Murphy and Brand [*^31^*](#_ENREF_31) was used to fit the *K_M_*, *n*, and *β* parameters in the flux expression for Complex IV. The reported fraction of c^2+^ was converted to concentration using the measurements given in Estabrook and Holowinsky*^[32](#_ENREF_32)^*.

*Proton leak*

The electrophoretically driven proton uptake via leak pathways was updated to account for the highly non-linear nature of the leak across energy-transducing membranes [*^33^*](#_ENREF_33). X_Hleak_ was set to 3.72 x10^7^ nmol/mg/min/M.

$J_{Hleak}=X_{Hleak}\left([H^{+}]_{e}e^{\left(\frac{3F\Delta\Psi}{4RT}\right)}-[H^{+}]_{m}e^{-\left(\frac{F\Delta\Psi}{4RT}\right)}\right)$.

**Figure S3.2. Proton leak rates fit to experimental data.** Respiration rate and membrane potential in the leak state from rat liver mitochondria was measured by incubating mitochondria in saturating glutamate and malate and titrating the membrane potential with potassium cyanide. For details see Porter et al.[*^34^*](#_ENREF_34).

S3B - Oxidative Phosphorylation Related Reactions Rates

*Adenine nucleotide translocase (ANT)*

The biochemical equation for ANT is defined as

$ATP_{x}+ADP_{e}\rightleftarrows ATP_{e}+ADP_{x}$.

The rate expression used in the model is

$J_{\text{ANT}}=X_{\text{ANT}}\frac{k_{2}^{ANT}q\frac{[\text{ATP}^{4-}]_{x}[\text{ADP}^{3-}]_{e}}{K_{o}^{D}}-k_{3}^{ANT}\frac{[\text{ATP}^{4-}]_{e}[\text{ADP}^{3-}]_{x}}{K_{o}^{T}}}{\left(1+\frac{[\text{ATP}^{4-}]_{e}}{K_{o}^{T}}+\frac{[\text{ADP}^{3-}]_{e}}{K_{o}^{D}}\right)\left([\text{ATP}^{4-}]_{x}+q[\text{ADP}^{3-}]_{x}\right)}$,

where

$k_{2}^{ANT}=k_{2}^{ANT,o}e^{\left(-3\alpha_{1}-4\alpha_{2}+\alpha_{3}\right)F\Delta\Psi/RT}$,

$k_{3}^{ANT}=k_{3}^{ANT,o}e^{\left(-4\alpha_{1}-3\alpha_{2}+\alpha_{3}\right)F\Delta\Psi/RT}$,

$K_{o}^{D}=K_{o}^{D,o}e^{3\delta_{D}RT/F\Delta\Psi}$,

$K_{o}^{T}=K_{o}^{\mathrm{T},o}e^{4\delta_{T}RT/F\Delta\Psi}$,

and

$q=\frac{k_{3}^{ANT}K_{o}^{D}}{k_{2}^{ANT}K_{o}^{T}}e^{F\Delta\Psi/RT}$.

**Table S3.5.** Adenine Nucleotide Translocase Parameters^a^

| Parameter | Definition | Value | Reference |
| --- | --- | --- | --- |
| *E_totANT_* | Total ANT content | 57.2 nmol/mg | b |
| $k_{2}^{ANT,o}$ | Forward translocation rate | 636 min^-1^ | [*^30^*](#_ENREF_30) |
| $k_{3}^{ANT,o}$ | Reverse translocation rate | 1.74E+3 min^-1^ | [*^30^*](#_ENREF_30) |
| $K_{o}^{D,o}$ | ADP binding constant | 1.5021 x10^-005^ M | [*^30^*](#_ENREF_30) |
| $K_{o}^{T,o}$ | ATP binding constant | 1.5021 x10^-005^ M | [*^30^*](#_ENREF_30) |
| *α_1_* | Translocation displacement constant | 0.2741unitless | [*^30^*](#_ENREF_30) |
| *α_2_* | Translocation displacement constant | 0.1926 unitless | [*^30^*](#_ENREF_30) |
| *α_3_* | Translocation displacement constant | -0.2397 unitless | [*^30^*](#_ENREF_30) |
| *δ_T_* | ATP displacement binding constant | 0.0459 unitless | [*^30^*](#_ENREF_30) |
| *δ_D_* | ADP displacement binding constant | -0.0088 unitless | [*^30^*](#_ENREF_30) |

^a^ Updated from Metelkin et al.[*^35^*](#_ENREF_35).

^b^ Adjustable parameter fit from data in Vinnakota et al.[*^14^*](#_ENREF_14).

*F_1_F_O_ ATP synthase*

The biochemical equation for F_1_F_O_ ATP synthase is defined as

$ADP_{m}+Pi_{m}\rightleftarrows ATP_{m}$.

The equilibrium constant is defined as

$K_{eq}=e^{-\left(\Delta_{r}G_{F_{1}F_{O}}^{0}+3F\Delta\Psi\right)/RT}\left(\frac{[H^{+}]_{e}^{nH}}{[H^{+}]_{x}^{nH-1}}\right)\left(\frac{P_{ATP}}{P_{ADP}P_{Pi}}\right)$.

The rate expression used in the model is

$J_{F_{1}F_{O}}=X_{F_{1}F_{O}}\left([ADP]_{x}[Pi]_{x}K_{eq}-[ATP]_{x}\right)$.

**Table S3.6.** F_1_F_O_ ATP synthase Parameters

| Parameter | Definition | Value | Reference |
| --- | --- | --- | --- |
| *X_F1FO_* | F_1_F_O_ activity | 7.5 X10^07^ nmol/M/min/mg | [*^10^*](#_ENREF_10)^a^ |
| $\Delta_{r}G_{F_{1}F_{O}}^{0}$ | Gibb’s free energy of reaction | -4.51 kJ/mol | [*^30^*](#_ENREF_30) |
| *nH* | H^+^:ATP ratio | 8/3 | [*^36^*](#_ENREF_36) |

^a^ The activity was lowered from the cited value to reduced the stiffness of the system of DAEs. This resulted in negligible differences in state variable dynamics for the simulations that were used to identify this parameter value.

*Inorganic phosphate carrier (PiC)*

The biochemical equation for the PiC is defined as

$Pi_{e}+H_{e}^{+}\rightleftarrows Pi_{x}+H_{x}^{+}$.

The rate expression used in the model is

$J_{\text{PiC}}=X_{PiC}([H^{+}]_{e}A-[H^{+}]_{x}P)/k_{PiC}/(1+A/k_{PiC})/(1+P/k_{PiC})$.

A is calculated as

$$A=[Pi]_{e}/K_{Pi}^{H}/P_{Pi_{e}}$$

P is calculated as

$$P=[Pi]_{x}[H^{+}]_{x}/K_{Pi}^{H}/P_{pi_{x}}$$

**Table S3.7** Inorganic Phosphate Parameters

| Parameter | Definition | Value | Reference |
| --- | --- | --- | --- |
| *X_PiC_* | PiC activity | 3.08x10^12^ nmol/min/mg | [*^10^*](#_ENREF_10) |
| *k_PiC_* | Pi binding constant | 1.61 mM | [*^10^*](#_ENREF_10) |

*ATPase*

The biochemical equation for an ATPase is defined as

$ATP_{e}\rightleftarrows ADP_{e}+Pi_{e}$.

The equilibrium constant is defined as

$K_{eq}=e^{-\left(\Delta_{r}G_{ATPase}^{0}/RT\right)}\left([H^{+}]_{e}^{-1}\right)\left(\frac{P_{ADP}P_{Pi}}{P_{ATP}}\right)$.

The rate expression used in the model is

$J_{ATPase}=X_{ATPase}/\left(1+[ADP]_{e}/K_{i,ADP}\right)\left(1-[ADP]_{e}[Pi]_{e}/[ATP]_{e}/K_{eq}\right)$.

**Table S3.8.** ATPase Parameters

| Parameter | Definition | Value | Reference |
| --- | --- | --- | --- |
| *X_ATPase_* | ATPase activity | 50 µM/min | a |
| $\Delta_{r}G_{ATPase}^{0}$ | Gibb’s free energy of reaction | 4.51 kJ/mol | [*^30^*](#_ENREF_30) |
| $K_{i,ADP}$ | ADP inhibition constant | 262 µM | b |

^a^ The value was based the steady state respiration rate after a bolus of ADP (i.e., ATP cycling state).

^b^ Adjustable parameter fit from data in Vinnakota et al.[*^14^*](#_ENREF_14).

S3D - Cation Related Reaction Rates

*Potassium-hydrogen exchanger*

The biochemical equation for KHE is defined as

$K_{m}^{+}+H_{e}^{+}\rightleftarrows K_{e}^{+}+H_{m}^{+}$.

The rate expression used in the model is

$$J_{KHE}=X_{KHE}\left([K^{+}]_{m}[H^{+}]_{e}-[K^{+}]_{e}[H^{+}]_{m}\right)$$

where *X_KHE_* is set to 2.0x10^10^ mol/min/mg.

*Calcium uniporter*

For the calcium uniporter, we opted to use as simple of a model as possible to explain the data. This model is derived assuming the energy barrier is centered in the membrane with a second allosteric calcium cooperative binding constant equal to the channel binding constant.

The biochemical equation for the MCU is defined as

$Ca_{e}^{2+}\rightleftarrows Ca_{m}^{2+}$.

The rate expression used in the model is

$$J_{\text{CU}}=X_{CU}\frac{(e^{(2\Delta\psi F/RT/2)}([Ca^{2+}]_{e}/K_{Ca})^{2}-e^{(-2\Delta\psi F/RT/2)}([Ca]_{x}/K_{Ca})^{2})}{1+([Ca]_{e}/K_{Ca})^{2}+([Ca]_{x}/K_{Ca})^{2}}$$

**Table S3.9** Mitochondrial Calcium Uniporter Parameters

| Parameter | Definition | Value | Reference |
| --- | --- | --- | --- |
| *X_MCU_* | MCU activity | 3.96E+5 nmol/min/mg | a |
| *K_Ca_* | Ca^2+^ binding constant | 4.8 mM | a |

^a^ Fit by simulating the experimental data sets described in the main paper.

**Sodium-calcium exchanger**

In order to balance Ca^2+^ influx from the calcium uniporter, mitochondria utilize what is known as the sodium-calcium exchanger (NCLX). The exchanger stoichiometry is assumed to be 3:1 resulting in an electrogenic exchange of three Na^+^ for one Ca^2+^. The expression is based on a rapid equilibrium random bi-bi mechanism and is similar to the Bazil et al. expression[*^4^*](#_ENREF_4) with no Ca^2+^-activation. The parameter values used for the rate expression are found in Table S3.10.

The biochemical equation for the NCLX is defined as

$Ca_{x}^{2+}+3Na_{e}^{+}\rightleftarrows Ca_{e}^{2+}+3Na_{x}^{+}$.

The rate expression used in the model is

$$J_{NCLX}=X_{NCLX}\frac{\frac{[Na^{+}]_{e}^{3}}{K_{Na}^{3}}\frac{[Ca^{2+}]_{x}}{K_{Ca}}e^{\frac{0.5\Delta\Psi F}{RT}}-\frac{[Na^{+}]_{x}^{3}}{K_{Na}^{3}}\frac{[Ca^{2+}]_{e}}{K_{Ca}}e^{-\frac{0.5\Delta\Psi F}{RT}}}{\left[\begin{matrix} 1+\frac{[Na^{+}]_{e}^{3}}{K_{Na}^{3}}+\frac{[Ca^{2+}]_{x}}{K_{Ca}}+\frac{[Na^{+}]_{e}^{3}}{K_{Na}^{3}}\frac{[Ca^{2+}]_{x}}{K_{Ca}}+ \\ \frac{[Na^{+}]_{x}^{3}}{K_{Na}^{3}}+\frac{[Ca^{2+}]_{e}}{K_{Ca}}+\frac{[Na^{+}]_{x}^{3}}{K_{Na}^{3}}\frac{[Ca^{2+}]_{e}}{K_{Ca}} \end{matrix}\right]}$$

**Table S3.10.** Sodium-Calcium Exchanger Parameters

| Parameter | Definition | Value | Reference |
| --- | --- | --- | --- |
| *X_NCLX_* | Sodium/Calcium exchanger activity | 104 nmol/min/mg | a |
| *K_Na_* | Sodium binding constant | 8 mM | [*^37^*](#_ENREF_37) |
| *K_Ca_* | Calcium binding constant | 240 µM | a |

^a^ Fit to simulation using the experimental data sets described in the main paper.

**Sodium-hydrogen exchanger**

The balance of sodium is achieved via the sodium-hydrogen exchanger. This exchanger is modeled similarly to sodium-calcium exchanger in that it assumes a rapid equilibrium random bi-bi mechanism except for an additional dependence on matrix pH as in Nguyen et al. [*^38^*](#_ENREF_38). The proton regulatory binding constant was refit to the data presented in Kapus et al. [*^39^*](#_ENREF_39). This rate was modified slightly since a better fit to the data was achieved by including a Hill coefficient of 2 in the regulatory binding term. The parameter values used for the rate expression are found in Table S3.26.

The biochemical equation for the NHE is defined as

$Na_{x}^{+}+H_{e}^{+}\rightleftarrows Na_{e}^{+}+H_{x}^{+}$.

The rate expression for mitochondrial Na^+^/H^+^ exchange is,

$$J_{NHE}=X_{NHE}\frac{[H^{+}]_{x}^{2}}{[H^{+}]_{x}^{2}+K_{H,reg}^{2}}\frac{\frac{[H^{+}]_{e}}{K_{H}}\frac{[Na^{+}]_{x}}{K_{Na}}-\frac{[H^{+}]_{x}}{K_{H}}\frac{[Na^{+}]_{e}}{K_{Na}}}{\left[\begin{matrix} 1+\frac{[H^{+}]_{e}}{K_{H}}+\frac{[Na^{+}]_{x}}{K_{Na}}+\frac{[H^{+}]_{e}}{K_{H}}\frac{[Na^{+}]_{x}}{K_{Na}}+ \\ \frac{[H^{+}]_{x}}{K_{H}}+\frac{[Na^{+}]_{e}}{K_{Na}}+\frac{[H^{+}]_{x}}{K_{H}}\frac{[Na^{+}]_{e}}{K_{Na}} \end{matrix}\right]}$$

**Table S3.26.** Sodium-Hydrogen Exchanger Parameters

| Parameter | Definition | Value | Reference |
| --- | --- | --- | --- |
| *X_NHE_* | Sodium/Hydrogen exchanger | 9.45E+5 nmol/min/mg | a |
| *K_Na_* | Sodium binding constant | 24.3 mM | [*^38^*](#_ENREF_38) |
| *K_H_* | Proton binding constant | 10^-8.5^ M | [*^38^*](#_ENREF_38) |
| *K_H,reg_* | Proton regulatory binding constant | 10^-7.2^ M | [*^39^*](#_ENREF_39)^b^ |

^a^ Fit to simulation using the experimental data sets described in the main paper.

^b^ Fit to data presented in the reference.

S4E – ROS Scavenging Related Reactions Rates

*Mangenese Superoxide Dismutase (MnSOD)*

The biochemical equation for MnSOD is defined as

$2O_{2}^{.-}+2H^{+}\rightleftarrows O_{2}+H_{2}O_{2}$.

The rate expression used in the model is

$$J_{SODx}=X_{SOD}[O_{2}^{.-}]_{x}$$

and

$$J_{SODe}=X_{SOD}[O_{2}^{.-}]_{e}$$

where the subscripts *x* and *e* denote matrix and extra-mitochondrial, respectively, *X_SOD_* is set to 2.0x10^4^ s^-1^ based on 10 µM [MnSOD] and a second order rate constant of 2.0x10^9^ M^-1^s^-1^ as given in [*^40^*](#_ENREF_40)*,* [*^41^*](#_ENREF_41). For simplicity, the extra-mitochondrial activity was set equal to the mitochondrial activity.

*H_2_O_2_ Reduction via GSH and Trx Pathways*

The biochemical equation for H_2_O_2_ scavenging is defined as

$H_{2}O_{2}+2H^{+}\rightleftarrows2H_{2}O$.

The rate expression used in the model is

$$J_{H2O2}=X_{H2O2}[H_{2}O_{2}]_{x}$$

where *X_H2O2_* is set to 1.32x10^4^ s^-1^ based on 60 µM [Prx3] and a second order rate constant of 2.0x10^7^ M^-1^s^-1^ and 2 µM [Gpx1] and a second order rate constant of 6.0x10^7^ M^-1^s^-1^ as given in [*^40^*](#_ENREF_40)*,* [*^41^*](#_ENREF_41).

The expression for H_2_O_2_ permeation through the mitochondrial membrane is

$$J_{H2O2perm}=k_{perm}\left([\mathrm{H}_{2}\mathrm{O}_{2}]_{x}-[\mathrm{H}_{2}\mathrm{O}_{2}]_{e}\right)$$

where *k_perm_* is set to 333 s^-1^ based on a membrane permeability coefficient of 2 µm/s [*^42^*](#_ENREF_42) and a membrane thickness of 6 nm.

**Figure S3.3. Calcium inhibition on ADP-stimulated respiration is independent of calpain activity even with longer calpain inhibitor incubation times.** Conditions are like those given in Fig 2 of the main paper except that the total calpain inhibitor incubation time was increased to 15 minutes. Briefly, 0.1 mg/ml mitochondria are incubated in the presence of 10 µM calpain inhibitor and substrates for five minutes before a bolus of CaCl_2_ is added followed by bolus of 500 µM ADP ten minutes after the CaCl_2_ bolus. Calpain inhibitors show no effect on the calcium-dependent inhibition of ADP-stimulated respiration. In all experiments, data are presented as mean +/- 95% confidence intervals. Individual data are presented as gray dots. There were no statistically significant differences within calcium treatment groups at a 0.05 alpha level. Control data for each calpain inhibitor were combined.

**S4: Model Code Details**

Simulate_Vinnakota_2016.m – script used to call the necessary functions to simulate the model and reproduce the plots for the Vinnakota et al. data set as given in the main article.

Vinnakota_2016.m – function that simulates the experimental protocol given in Vinnakota et al.

Calcium_Inhibition.m – function that simulates the experimental protocol for the pyruvate/malate experiments given in the main manuscript..

Calcium_Inhibition_SRot.m – function that simulates the experimental protocol for the succinate/rotenone experiments given in the main manuscript.

Simulate_Calcium_Inhibition.m – script used to call the necessary functions to simulate the model and reproduce the plots for the calcium inhibition data set as given in the main article.

DAEs.m – a function that defines the right-hand side of the system of differential algebraic equations that comprise the model.

Vinnakota_2016_Data.mat – cell array of experimental data for the Vinnakota et al. data set.

Calcium_Inhibition_Data.mat – cell array of experimental data for the calcium inhibition data set given in the main paper.

Model_Parameters.mat – structure containing model parameters.

**References**

[1] Bazil, J. N., Beard, D. A., and Vinnakota, K. C. (2016) Catalytic Coupling of Oxidative Phosphorylation, ATP Demand, and Reactive Oxygen Species Generation, *Biophysical journal* *110*, 962-971.

[2] Bazil, J. N. (2017) Analysis of a Functional Dimer Model of Ubiquinol Cytochrome c Oxidoreductase, *Biophysical journal* *113*, 1599-1612.

[3] Bazil, J. N., Blomeyer, C. A., Pradhan, R. K., Camara, A. K., and Dash, R. K. (2013) Modeling the calcium sequestration system in isolated guinea pig cardiac mitochondria, *J Bioenerg Biomembr* *45*, 177-188.

[4] Bazil, J. N., Buzzard, G. T., and Rundell, A. E. (2010) Modeling mitochondrial bioenergetics with integrated volume dynamics, *PLoS computational biology* *6*, e1000632.

[5] Smith, R. M., Martell, A. E., and Chen, Y. (1991) Critical-Evaluation of Stability-Constants for Nucleotide Complexes with Protons and Metal-Ions and the Accompanying Enthalpy Changes, *Pure Appl Chem* *63*, 1015-1080.

[6] Smith, R. M., and Alberty, R. A. (1956) The Apparent Stability Constants of Ionic Complexes of Various Adenosine Phosphates with Monovalent Cations, *J Phys Chem-Us* *60*, 180-184.

[7] O'Sullivan, W. J., and Smithers, G. W. (1979) Stability constants for biologically important metal-ligand complexes, *Methods Enzymol* *63*, 294-336.

[8] Qi, F., Chen, X., and Beard, D. A. (2008) Detailed kinetics and regulation of mammalian NAD-linked isocitrate dehydrogenase, *Biochim Biophys Acta* *1784*, 1641-1651.

[9] Magnus, G., and Keizer, J. (1997) Minimal model of beta-cell mitochondrial Ca2+ handling, *Am J Physiol* *273*, C717-733.

[10] Beard, D. A. (2005) A biophysical model of the mitochondrial respiratory system and oxidative phosphorylation, *PLoS computational biology* *1*, e36.

[11] Li, X., Wu, F., Qi, F., and Beard, D. A. (2011) A database of thermodynamic properties of the reactions of glycolysis, the tricarboxylic acid cycle, and the pentose phosphate pathway, *Database : the journal of biological databases and curation* *2011*, bar005.

[12] Vinnakota, K. C., Wu, F., Kushmerick, M. J., and Beard, D. A. (2009) Multiple ion binding equilibria, reaction kinetics, and thermodynamics in dynamic models of biochemical pathways, *Methods in enzymology* *454*, 29-68.

[13] Alberty, R. A. (2003) *Thermodynamics of biochemical reactions*, Wiley-Interscience, Hoboken, N.J.

[14] Vinnakota, K. C., Bazil, J. N., Van den Bergh, F., Wiseman, R. W., and Beard, D. A. (2016) Feedback Regulation and Time Hierarchy of Oxidative Phosphorylation in Cardiac Mitochondria, *Biophysical journal* *110*, 972-980.

[15] Bazil, J. N., Pannala, V. R., Dash, R. K., and Beard, D. A. (2014) Determining the origins of superoxide and hydrogen peroxide in the mammalian NADH:ubiquinone oxidoreductase, *Free radical biology & medicine* *77*, 121-129.

[16] Sled, V. D., Rudnitzky, N. I., Hatefi, Y., and Ohnishi, T. (1994) Thermodynamic analysis of flavin in mitochondrial NADH:ubiquinone oxidoreductase (complex I), *Biochemistry* *33*, 10069-10075.

[17] Ingledew, W. J., and Ohnishi, T. (1980) An analysis of some thermodynamic properties of iron-sulphur centres in site I of mitochondria, *The Biochemical journal* *186*, 111-117.

[18] Brandt, U., and Okun, J. G. (1997) Role of deprotonation events in ubihydroquinone:cytochrome c oxidoreductase from bovine heart and yeast mitochondria, *Biochemistry* *36*, 11234-11240.

[19] Rich, P. R., Jeal, A. E., Madgwick, S. A., and Moody, A. J. (1990) Inhibitor effects on redox-linked protonations of the b haems of the mitochondrial bc1 complex, *Biochim Biophys Acta* *1018*, 29-40.

[20] Crofts, A. R., Shinkarev, V. P., Kolling, D. R., and Hong, S. (2003) The modified Q-cycle explains the apparent mismatch between the kinetics of reduction of cytochromes c1 and bH in the bc1 complex, *J Biol Chem* *278*, 36191-36201.

[21] Ohnishi, T., and Trumpower, B. L. (1980) Differential effects of antimycin on ubisemiquinone bound in different environments in isolated succinate . cytochrome c reductase complex, *J Biol Chem* *255*, 3278-3284.

[22] Glaser, E. G., Meinhardt, S. W., and Crofts, A. R. (1984) Reduction of cytochrome b-561 through the antimycin-sensitive site of the ubiquinol-cytochrome c2 oxidoreductase complex of Rhodopseudomonas sphaeroides, *FEBS Lett* *178*, 336-342.

[23] Glaser, E. G., and Crofts, A. R. (1984) A new electrogenic step in the ubiquinol:cytochrome c2 oxidoreductase complex of Rhodopseudomonas sphaeroides, *Biochim Biophys Acta* *766*, 322-333.

[24] Robertson, D. E., and Dutton, P. L. (1988) The nature and magnitude of the charge-separation reactions of ubiquinol cytochrome c2 oxidoreductase, *Biochim Biophys Acta* *935*, 273-291.

[25] Ding, H., Moser, C. C., Robertson, D. E., Tokito, M. K., Daldal, F., and Dutton, P. L. (1995) Ubiquinone pair in the Qo site central to the primary energy conversion reactions of cytochrome bc1 complex, *Biochemistry* *34*, 15979-15996.

[26] Wikström, M., and Royal Society of Chemistry (Great Britain) (2005) *Biophysical and structural aspects of bioenergetics*, Royal Society of Chemistry, Cambridge, UK.

[27] Lass, A., Agarwal, S., and Sohal, R. S. (1997) Mitochondrial ubiquinone homologues, superoxide radical generation, and longevity in different mammalian species, *J Biol Chem* *272*, 19199-19204.

[28] Schwerzmann, K., Cruz-Orive, L. M., Eggman, R., Sanger, A., and Weibel, E. R. (1986) Molecular architecture of the inner membrane of mitochondria from rat liver: a combined biochemical and stereological study, *J Cell Biol* *102*, 97-103.

[29] Kuo-chen, C., and Shou-ping, J. (1974) Studies on the rate of diffusion-controlled reactions of enzymes. Spatial factor and force field factor, *Sci Sin* *27*, 664-680.

[30] Wu, F., Zhang, E. Y., Zhang, J., Bache, R. J., and Beard, D. A. (2008) Phosphate metabolite concentrations and ATP hydrolysis potential in normal and ischaemic hearts, *The Journal of physiology* *586*, 4193-4208.

[31] Murphy, M. P., and Brand, M. D. (1987) The control of electron flux through cytochrome oxidase, *The Biochemical journal* *243*, 499-505.

[32] Estabrook, R. W., and Holowinsky, A. (1961) Studies on the content and organization of the respiratory enzymes of mitochondria, *The Journal of biophysical and biochemical cytology* *9*, 19-28.

[33] Garlid, K. D., DiResta, D. J., Beavis, A. D., and Martin, W. H. (1986) On the mechanism by which dicyclohexylcarbodiimide and quinine inhibit K+ transport in rat liver mitochondria, *J Biol Chem* *261*, 1529-1535.

[34] Porter, R. K., Joyce, O. J., Farmer, M. K., Heneghan, R., Tipton, K. F., Andrews, J. F., McBennett, S. M., Lund, M. D., Jensen, C. H., and Melia, H. P. (1999) Indirect measurement of mitochondrial proton leak and its application, *Int J Obes Relat Metab Disord* *23 Suppl 6*, S12-18.

[35] Metelkin, E., Goryanin, I., and Demin, O. (2006) Mathematical modeling of mitochondrial adenine nucleotide translocase, *Biophysical journal* *90*, 423-432.

[36] Nicholls, D. G., and Ferguson, S. J. (2013) *Bioenergetics*, Fourth edition / ed., Academic Press, Elsevier, Amsterdam.

[37] Paucek, P., and Jaburek, M. (2004) Kinetics and ion specificity of Na(+)/Ca(2+) exchange mediated by the reconstituted beef heart mitochondrial Na(+)/Ca(2+) antiporter, *Biochim Biophys Acta* *1659*, 83-91.

[38] Nguyen, M. H., Dudycha, S. J., and Jafri, M. S. (2007) Effect of Ca2+ on cardiac mitochondrial energy production is modulated by Na+ and H+ dynamics, *Am J Physiol Cell Physiol* *292*, C2004-2020.

[39] Kapus, A., Ligeti, E., and Fonyo, A. (1989) Na+/H+ exchange in mitochondria as monitored by BCECF fluorescence, *FEBS Lett* *251*, 49-52.

[40] Murphy, M. P. (2012) Mitochondrial thiols in antioxidant protection and redox signaling: distinct roles for glutathionylation and other thiol modifications, *Antioxidants & redox signaling* *16*, 476-495.

[41] Murphy, M. P. (2009) How mitochondria produce reactive oxygen species, *The Biochemical journal* *417*, 1-13.

[42] Bienert, G. P., Schjoerring, J. K., and Jahn, T. P. (2006) Membrane transport of hydrogen peroxide, *Biochim Biophys Acta* *1758*, 994-1003.
